# The Exact Hypergeometric Posterior Method for Accurate Inference of Population Size from Mark–Recapture Data

**DOI:** 10.1007/s11538-026-01687-3

**Published:** 2026-06-25

**Authors:** Danial Mirzaee, Seyed Amir Malekpour, Ata Kalirad

**Affiliations:** 1 Department of Biology, University of Tehran, Tehran, 14155-6455 Iran; 2 School of Biological Sciences, Institute for Research in Fundamental Sciences (IPM), Tehran, Iran; 3 Department for Integrative Evolutionary Biology, Max Planck Institute for Biology, Tübingen, 72076 Germany

**Keywords:** Mark–recapture, Capture–recapture, Discrete Bayesian inference, Noncentral hypergeometric distribution

## Abstract

Reliable inference of census population size (*N*) from mark–recapture data is essential in ecology, conservation, and epidemiology, yet standard estimators often rely on asymptotic approximations that can misrepresent uncertainty. We introduce the Exact Hypergeometric Posterior (EHP), obtained by normalizing the hypergeometric likelihood for the classic two-sample design, which yields an exact posterior probability mass function over integer *N*. Closed-form normalization enables rapid evaluation of posterior summaries and exact highest-posterior-density (HPD) credible intervals. In this study, we show how heavy right tails naturally arise in sparse-recapture regimes and provide a principled truncation using an upper bound *K*, such as a plausible carrying capacity or sampling-frame limit, to regularize inference when needed. For repeated sampling, independent events can be combined by posterior multiplication and renormalization, yielding substantial gains in precision without large-sample approximations. Additionally, we extend the likelihood to account for loss of marked individuals and differential catchability by introducing an availability (retention) term $\phi (t)$ and a relative catchability parameter $\omega$ via Fisher’s noncentral hypergeometric model. Simulations and empirical examples confirm close agreement between analytic posteriors and Monte Carlo reference posteriors using our method. Together, these results provide a transparent, computationally tractable framework for finite-sample population-size inference in closed and partially open systems.

## Introduction

Census population size (*N*, i.e., the number of individuals in a population at a given time and place) and effective population size ($N_e$, i.e., the size of an idealized population experiencing the same magnitude of genetic drift and inbreeding as the focal population) (Wright 1931) are major determinants of evolutionary outcomes. Variation in population size shapes processes on the microevolutionary scale, including the expansion and contraction of genomes, the accumulation of genetic incompatibilities, and the evolution of mutation rate, and it can also influence macroevolutionary events such as speciation and extinction (Marzluff and Dial 1991; Dennis 2002; Lynch and Conery 2003; Kalirad et al. 2024). In most natural populations, *N* and $N_e$ are not equal, with $N_e$ often substantially smaller than *N*, and $N_e$ can be inferred from neutral genetic variation (Frankham 2007; Wang 2005; Charlesworth 2009). However, estimating *N* itself remains valuable. In particular, the relationship between *N* and $N_e$ can reveal key demographic and reproductive features of a species, and *N* can provide a practical basis for approximating $N_e$ in applied settings (Waples 2024).

Among the many approaches for estimating census population size, mark–recapture (MR) has become a standard tool in ecology and epidemiology (Jolly 1965; Seber 1986; Chao et al. 2001; Seber and Schofield 2019). In conservation biology, management decisions often depend on whether a population exceeds minimum thresholds needed to avoid extinction and to preserve adaptive potential, which in turn requires reliable estimates of both *N* and $N_e$ (Traill et al. 2010; Jamieson and Allendorf 2012). In epidemiology, capture–recapture methods are used when surveillance is incomplete and cases (or at-risk individuals) are recorded across multiple, partially overlapping data sources. In such contexts, MR can help estimate total burden (including unobserved cases) and support planning, targeting, and evaluation of interventions (Thandrayen and Baffour 2024; Rerolle et al. 2024).

Classical MR estimators rely on a hypergeometric or binomial sampling model for the number of marked individuals recaptured on a second visit. These assumptions, and the resulting estimators, have been critiqued for limited accuracy in sparse-data regimes and for sensitivity to ecological violations of closure and equal catchability (Dettloff 2023; Seber and Schofield 2019). A variety of modifications have been proposed to address these issues, including Pollock’s robust design, which reduces the impact of capture heterogeneity on abundance estimates (Pollock 1982), and estimators tailored to sparse recapture data (Chao 1989). For the Lincoln–Petersen estimator, Chapman’s adjustment offers an approximately unbiased point estimate of *N* and admits an approximate variance formula (Chapman 1951). Confidence intervals (CIs) are commonly constructed using normal-approximation methods applied to a log-transformed estimator, but such intervals can exhibit poor coverage when sample sizes are small or recapture probabilities are low (Otis et al. 1978). Alternative approaches include exact CIs based on binomial inversion (Clopper and Pearson 1934), bootstrap resampling techniques (Buckland and Garthwaite 1991), and likelihood-ratio procedures that invert the profile likelihood for *N* (Gimenez et al. 2005).

Beyond simple two-sample designs, inference for mark–recapture data often uses full-likelihood constructs under closed-population assumptions. Capture histories are modeled with a multinomial likelihood parameterized by capture probabilities; maximum-likelihood estimation provides point estimates of *N* and capture parameters, and uncertainty is sometimes summarized using asymptotic standard errors and Wald-type intervals (Gimenez et al. 2005). However, such intervals can be unreliable when likelihoods are asymmetric, sample sizes are small, or estimates lie near a boundary. For this reason, profile-likelihood confidence intervals have been advocated as a more appropriate likelihood-based alternative for capture–recapture estimators (Evans et al. 1996; O’Brien and Silcox 2024). Bayesian formulations can incorporate prior knowledge and compute highest-posterior-density (HPD) intervals, often via Markov chain Monte Carlo (MCMC) (Webster and Kemp 2013; Gazey and Staley 1986). Nonetheless, key challenges persist across approaches. Violations of closure, heterogeneity in capture probability, and behavioral responses to trapping can induce bias in both point estimates and interval coverage (Dettloff 2023; Pledger and Efford 1998; Gimenez et al. 2018).

Here we develop the Exact Hypergeometric Posterior (EHP) framework to supplement the classic two-sample MR design. We derive an exact posterior mass function for population size *N* by normalizing the hypergeometric likelihood, and we show that posterior summaries and discrete HPD credible intervals follow directly from this distribution. We then extend the baseline model in three directions motivated by applied monitoring: (i) truncation at an ecological upper bound *K* to incorporate carrying-capacity or sampling-frame constraints, (ii) joint inference across repeated or independent sampling events by multiplying event-specific posteriors over a shared population size, and (iii) likelihood-level treatment of departures from closure and equal catchability by modeling availability of marked individuals through a retention probability $\phi (t)$ (capturing mortality, permanent emigration, or tag loss) and differential catchability through an odds ratio $\omega$ using Fisher’s noncentral hypergeometric distribution. We validate the analytic posteriors by Monte Carlo simulation and compare interval behavior against widely used estimators and CI constructions, and use empirical examples to illustrate how ecological knowledge and auxiliary data can be incorporated transparently.

## Exact Hypergeometric Posterior (EHP) for Population Size Estimation

This section derives the exact hypergeometric posterior (EHP) for the census population size *N* from two-sample MR data under the classical closed-population sampling model. Rather than relying on a single point estimator, the EHP method yields an exact posterior probability mass function (PMF) over integer *N*, enabling exact posterior summaries (mode, median, mean when defined) and discrete HPD credible intervals. We then introduce a finite upper bound *K* to incorporate ecological constraints and to enable further calculations such as combining information across multiple independent MR events by multiplying event-specific posterior PMFs.

### Single-event EHP

**Fig. 1:**
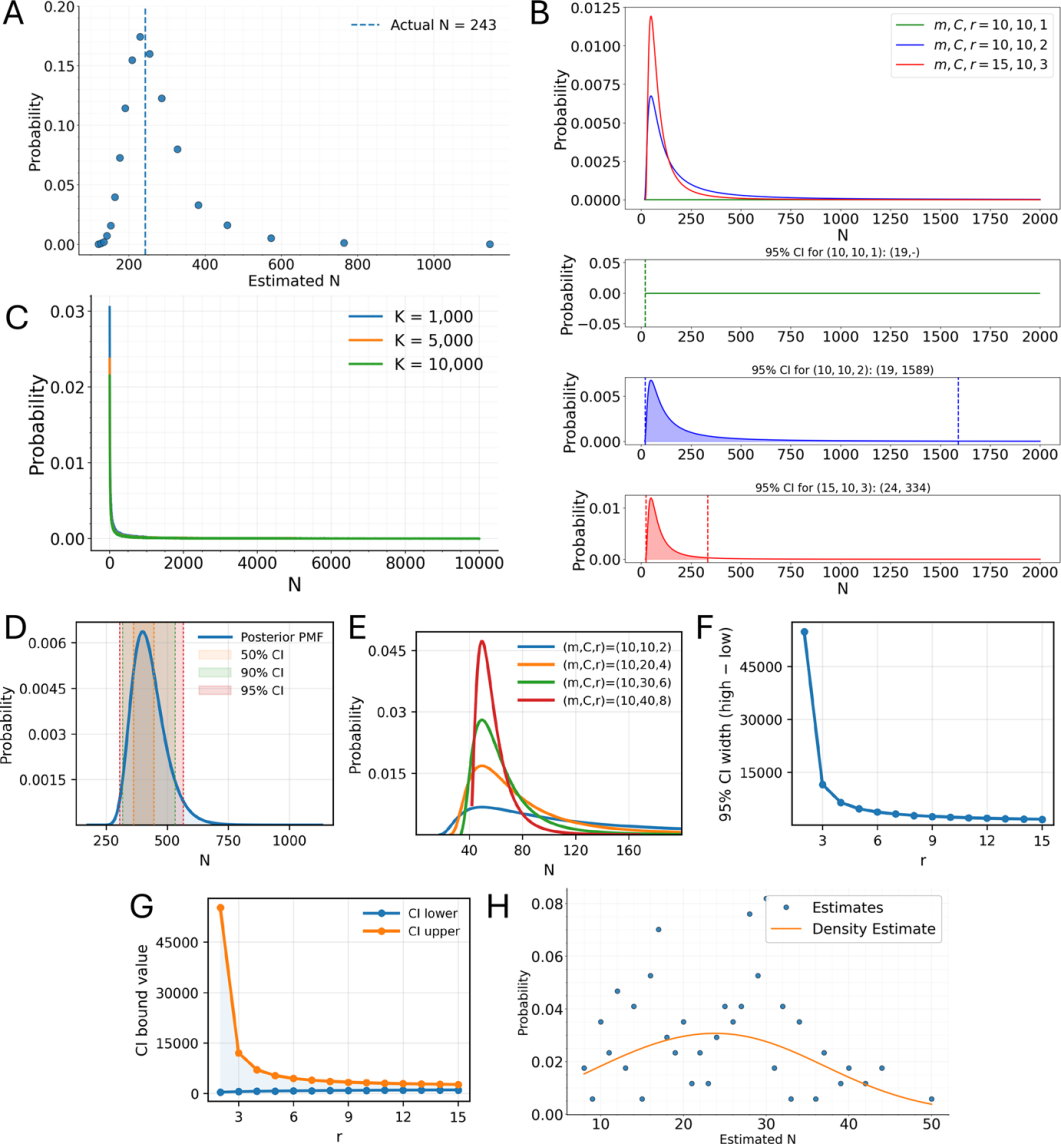
Exact hypergeometric posterior (EHP) for a single two-sample mark–recapture event. **(A)** Monte Carlo illustration of estimator variability under a known population size. We simulate many independent two-sample experiments with $N=243$, $m=81$, and $C=27$ under hypergeometric sampling; points show the hypergeometric distribution of the Lincoln–Petersen estimate across replicates (excluding $r=0$), and the dashed line marks the true $N$. **(B)** EHP distributions for very small recapture counts show how heavy right tails emerge as information decreases. The top panel shows representative (untruncated) EHP PMFs for $r\in \{1,2,3\}$ (datasets indicated in the legend); lower panels show the corresponding 95% HPD credible intervals. For $r=2$ and $r=3$ the posterior is proper but strongly right-skewed; for $r=1$ the unbounded normalization fails, so a finite upper bound is undefined without truncation. **(C)** Consequence of the $r=1$ divergence: simulated posteriors for an $r=1$ dataset under bounded support $N\in \{N_{\min },\ldots ,K\}$ depend on the chosen $K$ and do not converge as $K\rightarrow \infty$, motivating an ecological/sampling-frame upper bound (or informative prior) when recaptures are extremely sparse. **(D)** Example posterior ($m,C,r=100,100,25$) with nested 50%, 90%, and 95% HPD credible intervals, illustrating asymmetric mass accumulation and the dominant upper tail. **(E)** Equal point estimates can mask very different uncertainty: four datasets share the same Lincoln–Petersen ratio $mC/r$, yet larger $r$ yields a markedly more concentrated posterior PMF. **(F,G)** Posterior concentration as recapture information increases. In a controlled sequence where $r$ increases from 2 to 15 (fixed $m=150$, $C=10r$), the 95% HPD width contracts rapidly at small $r$ and then shows diminishing returns (F). The tightening is driven mainly by a sharp drop in the upper HPD bound, while the lower bound rises more gradually (G). **(H)** Empirical distribution of Lincoln–Petersen estimates $mC/r$ from a butterfly study, shown as a frequency distribution with a smooth density overlay. The right-skewed spread mirrors panels (E–G), illustrating heavy-tailed uncertainty under sparse recapture (Hinneberg et al. 2022)

Consider a two-sample MR study where, in the first sample, *m* individuals are captured, marked, and released. In the second sample, *C* individuals are captured, among which *r* are marked recaptures. Under the standard closed-population model (no births, deaths, immigration, or emigration between samples; marks are retained; and marked and unmarked individuals are equally catchable at the second occasion), the recapture count *r* follows the hypergeometric sampling distribution (Figure 1A) (Lincoln 1930; Petersen 1896; Dettloff 2023):

1 $$\begin{aligned} P(r \mid N) = \frac{\left( {\begin{array}{c}m\\ r\end{array}}\right) \left( {\begin{array}{c}N-m\\ C-r\end{array}}\right) }{\left( {\begin{array}{c}N\\ C\end{array}}\right) }, \quad r = 0,1,\ldots ,\min (m,C). \end{aligned}$$

For inference on *N* given observed (*m*, *C*, *r*), we treat *N* as the unknown integer parameter and normalize the likelihood over its admissible support. The smallest feasible population size is $N_{\min }=m+C-r$, the number of individuals observed at least once across the two samples. Adopting a discrete uniform prior on $N\in \{N_{\min },N_{\min }+1,\ldots \}$ (equivalently, normalizing the likelihood kernel), we define the EHP as

2 $$\begin{aligned} \begin{aligned} P(N \mid r)&= \frac{\displaystyle \frac{\left( {\begin{array}{c}m\\ r\end{array}}\right) \left( {\begin{array}{c}N-m\\ C-r\end{array}}\right) }{\left( {\begin{array}{c}N\\ C\end{array}}\right) }}{\displaystyle \sum _{x=m+C-r}^{\infty }\frac{\left( {\begin{array}{c}m\\ r\end{array}}\right) \left( {\begin{array}{c}x-m\\ C-r\end{array}}\right) }{\left( {\begin{array}{c}x\\ C\end{array}}\right) }} \quad ,\\ N&=m+C-r,\,m+C-r+1,\,m+C-r+2,\ldots \end{aligned} \end{aligned}$$

where *m* and *C* are treated as fixed by design and *r* is the observed data from the recapture sample.

The normalizing series in Eq. (2) can be evaluated in closed form. Writing the binomial coefficients in factorial form and shifting the index by $y=N-(m+C-r)$ gives

3 $$\begin{aligned} \sum _{y=0}^{\infty } \frac{(y+C-r)!\,(y+m-r)!}{y!\,(y+m+C-r)!}\times \frac{m!\,C!}{r!\,(m-r)!\,(C-r)!} \quad . \end{aligned}$$

A closed-form posterior PMF can be derived from Eq. 3 (see Appendix A for more detailed calculations):

4 $$\begin{aligned} \boxed { P(N \mid r)= \frac{\left( {\begin{array}{c}m\\ r\end{array}}\right) \left( {\begin{array}{c}N-m\\ C-r\end{array}}\right) }{\left( {\begin{array}{c}N\\ C\end{array}}\right) }\, \frac{r(r-1)}{mC} \quad \text {for } N\ge m+C-r \text { and } 1<r\le \min (m,C) \quad . } \end{aligned}$$

A key practical feature of Eq. (4) is its heavy-tail behavior for small *r*. As shown in Appendix B, the likelihood kernel decays as $P(r\mid N)\propto N^{-r}$ for large *N*, so the unbounded posterior is proper only when $r>1$ and becomes increasingly right-skewed as *r* decreases. Figure 1B illustrates this directly: small recapture counts produce broad posteriors with substantial right-tail mass, leading to very wide 95% intervals (and, without further regularization, effectively unbounded upper limits). In particular, when $r=1$, the unbounded posterior cannot be normalized, motivating the finite-*K* formulation in Section 2.2. Figure 1C visualizes this divergence: when $r=1$, increasing the truncation point *K* continues to reallocate probability mass to larger *N* (and inflates the upper interval bound), showing that the $K\rightarrow \infty$ normalization does not converge for the unbounded model.

The EHP distribution is unimodal for $r>1$, and its mode aligns closely with the classical Lincoln–Petersen point estimator. A convenient diagnostic is the ratio of successive posterior masses:

5 $$\begin{aligned} \frac{P(N+1\mid r)}{P(N\mid r)} = \frac{(N+1-m)(N+1-C)}{(N+1)\bigl (N+1-(m+C-r)\bigr )} \quad . \end{aligned}$$

Equation (5) increases above and then drops below 1 exactly once, implying a single peak (or a two-point plateau in the knife-edge case). Solving $\frac{P(N+1\mid r)}{P(N\mid r)} \le 1$ gives

$$ (N+1-m)(N+1-C)\le (N+1)\bigl (N+1-(m+C-r)\bigr ) \iff N+1 \ge \frac{mC}{r} \quad . $$

Therefore

- If $$\displaystyle \frac{mC}{r}\notin \mathbb {Z}$$:
  $$ \widehat{N}_{\textrm{mode}}=\left\lfloor \frac{mC}{r}\right\rfloor \quad . $$
- If $$\displaystyle \frac{mC}{r}\in \mathbb {Z}$$:
  $$ \widehat{N}_{\textrm{mode}}\in \left\{ \frac{mC}{r}-1,\ \frac{mC}{r}\right\} \quad , $$
  subject to the support lower bound (if $\displaystyle \frac{mC}{r}-1<N_{\min }$, then only $N_{\min }$ remains feasible).

When $r>2$, the EHP posterior mean exists and admits a simple closed form (see Appendix C):

6 $$\begin{aligned} \mathbb {E}[N\mid r] =\sum _{N=m+C-r}^{\infty } N\,P(N\mid r) =\frac{(m-1)(C-1)}{r-2} \quad , \qquad r>2. \end{aligned}$$

This quantity provides a natural point estimate of *N*. An equivalent Bayesian derivation of the Lincoln–Petersen posterior mean was given by (Webster and Kemp 2013) via a different route. For $r=2$, the posterior remains proper, but the mean diverges.

To report uncertainty, we compute discrete HPD credible intervals from the posterior PMF. Because the posterior is typically unimodal and asymmetric, HPD intervals provide an exact, compact summary of posterior mass. Throughout we report the shortest contiguous $(1-\alpha )$ interval

7 $$\begin{aligned} (L_\alpha ,U_\alpha )=\arg \min _{L\le U}\left\{ U-L:\sum _{N=L}^{U} P(N\mid r)\ge 1-\alpha \right\} \quad , \end{aligned}$$

which can be obtained efficiently by expanding outward from the mode and adding the larger of the two adjacent tail masses at each step. Alternatively, for truncated posteriors or in any case where unimodality is not guaranteed, an HPD set can be constructed by ranking *N* by posterior mass and accumulating to $1-\alpha$, reporting the minimum and maximum selected values as bounds (Appendix D). The *mode expansion* approach is the default method used throughout this study because of higher accuracy and stability. Figure 1D shows the nested 50%, 90%, and 95% HPD intervals on a representative EHP curve, highlighting how uncertainty expands from the core mass to the tails.

Figure 1E emphasizes that equal point estimates can mask very different uncertainty: the four example datasets share the same Lincoln–Petersen estimate *mC*/*r* but yield progressively more concentrated EHP distributions as sample sizes increase. This behavior is also reflected in Table 1, where credible intervals tighten markedly with increasing information. Increasing *r* rapidly reduces the width of the 95% HPD interval (Figure 1F), primarily by pulling down the upper bound while the lower bound increases more gradually (Figure 1G). Finally, Figure 1H provides an external validation of the trends in Figure 1E–G using a publicly available dataset, indicating that the spread of Lincoln–Petersen estimates and associated uncertainty follows the same qualitative EHP behavior, with intervals tightening rapidly as recapture information increases. Across these data, EHP point summaries can somewhat track classical estimators when recaptures are plentiful, but the implied uncertainty can differ substantially in sparse-recapture regimes. Table 1 gives representative single-event results and directly compares posterior summaries and HPD credible intervals to widely used estimators and confidence intervals (e.g., Lincoln–Petersen/Chapman point estimates and common CI constructions), highlighting that standard approximations can be overly symmetric and understate right-tail uncertainty, especially when recaptures are few. Conversely, when information is abundant (large samples and many recaptures), established interval constructions can be conservative, often producing slightly wider intervals than EHP and thus potentially overestimating uncertainty.

**Table 1:** Single-event EHP summaries and standard two-sample estimators. For each representative two-sample dataset (*m*, *C*, *r*) (first-sample marked *m*, second-sample catch *C*, and marked recaptures *r*), we report the EHP mode/mean/median and the 95% HPD credible interval for population size *N*, alongside the Lincoln–Petersen (Petersen 1896; Lincoln 1930) and Chapman point estimates (Chapman 1951; Dettloff 2023) and two 95% CIs (Chapman log-normal and exact hypergeometric inversion) (Adams 1951; Dang et al. 2021; Wang 2015).

(*m*, *C*, *r*)	Mode	Mean	Median	95% HPD credible interval	LP Est.	Chapman Est.	Chapman CI	Exact hypergeometric CI
$(100,\,10,\,1)$	–	–	–	–	1000.0	554.5	(200.9, 1530.4)	(229, 39552)
$(100,\,20,\,2)$	1000	–	2773	(239, 36746)	1000.0	706.0	(288.5, 1727.8)	(322, 8067)
$(100,\,30,\,3)$	999	2871.0	1736	(333, 7989)	1000.0	781.8	(350.3, 1744.5)	(386, 4702)
$(100,\,40,\,4)$	999	1930.5	1461	(399, 4655)	1000.0	827.2	(397.9, 1719.7)	(434, 3546)
$(100,\,50,\,5)$	999	1617.0	1334	(447, 3498)	1000.0	857.5	(436.3, 1685.3)	(472, 2969)
$(100,\,60,\,6)$	999	1460.3	1262	(486, 2923)	1000.0	879.1	(468.4, 1650.2)	(503, 2622)
$(100,\,70,\,7)$	999	1366.2	1215	(517, 2578)	1000.0	895.4	(495.7, 1617.2)	(530, 2390)
$(100,\,80,\,8)$	999	1303.5	1181	(544, 2348)	1000.0	908.0	(519.5, 1586.9)	(552, 2223)
$(100,\,90,\,9)$	999	1258.7	1157	(566, 2183)	1000.0	918.1	(540.5, 1559.5)	(572, 2097)
$(100,\,100,\,10)$	999	1225.1	1138	(586, 2059)	1000.0	926.4	(559.2, 1534.5)	(590, 1997)
$(100,\,500,\,50)$	999	1029.2	1019	(843, 1234)	1000.0	991.2	(826.7, 1188.3)	(840, 1242)
$(1000,\,1000,\,1000)$	1000	1000.0	1000	(1000, 1000)	1000.0	1000.0	(1000, 1000)	(1000, 1000)
$(200,\,200,\,20)$	1999	2200.1	2128	(1372, 3170)	2000.0	1922.9	(1322.3, 2796.1)	(1366, 3163)
$(500,\,500,\,50)$	4999	5187.5	5123	(3934, 6558)	5000.0	4920.6	(3854.5, 6281.5)	(3914, 6576)
$(1000,\,1000,\,100)$	10000	10183.7	10121	(8430, 12047)	10000.0	9919.8	(8331.4, 11811)	(8400, 12073)
$(10000,\,10000,$ 500)	199999	200763.1	200508	(184243, 217716)	200000.0	199639.7	(183721.1, 216937.7)	(184054, 217840)

Dashes indicate quantities that do not exist under unbounded support.

### Imposing an upper bound *K* on the population size

In many ecological settings, *N* is *a priori* bounded within the sampling frame by space, resources, and behavior (Berryman 2004). We incorporate such information by imposing a finite upper bound *K* on the support of *N*, yielding a truncated posterior PMF. Importantly, *K* is not restricted to spatially open systems with immigration and emigration. In a spatially closed population that nonetheless experiences demographic turnover (births and deaths), *K* can be interpreted as an upper bound on the *instantaneous census size* within the focal area at the time of sampling, arising from resource limits, nest-site availability, territoriality, or social structure (for example, a fenced reserve where group territories limit the maximum number of mammals) (Both and Visser 2003). In such applications, *K* functions as an explicit, system-specific constraint on plausible *N* values. When demographic turnover affects the availability of marked individuals between samples, it acts primarily through the recapture process (changing the distribution of *r*) (Schaub and Royle 2014; Fujiwara and Caswell 2002). That mechanism is handled by the extended recapture likelihood in Section 3, while *K* continues to serve as an upper-support constraint on *N*.

Formally, with *K* specified, we restrict normalization to $\{N_{\min },\ldots ,K\}$:

8 $$\begin{aligned} \boxed { P(N \mid r,K) = \frac{\displaystyle \frac{\left( {\begin{array}{c}m\\ r\end{array}}\right) \left( {\begin{array}{c}N-m\\ C-r\end{array}}\right) }{\left( {\begin{array}{c}N\\ C\end{array}}\right) }}{\displaystyle \sum _{x=m+C-r}^{K}\frac{\left( {\begin{array}{c}m\\ r\end{array}}\right) \left( {\begin{array}{c}x-m\\ C-r\end{array}}\right) }{\left( {\begin{array}{c}x\\ C\end{array}}\right) }} \qquad \text {for } N=m+C-r,\ldots ,K \quad . } \end{aligned}$$

The *K*-truncated posterior is always proper (including when $r=1$), and it reduces to the unbounded EHP when *K* is taken large relative to the posterior mass.

**Fig. 2:**
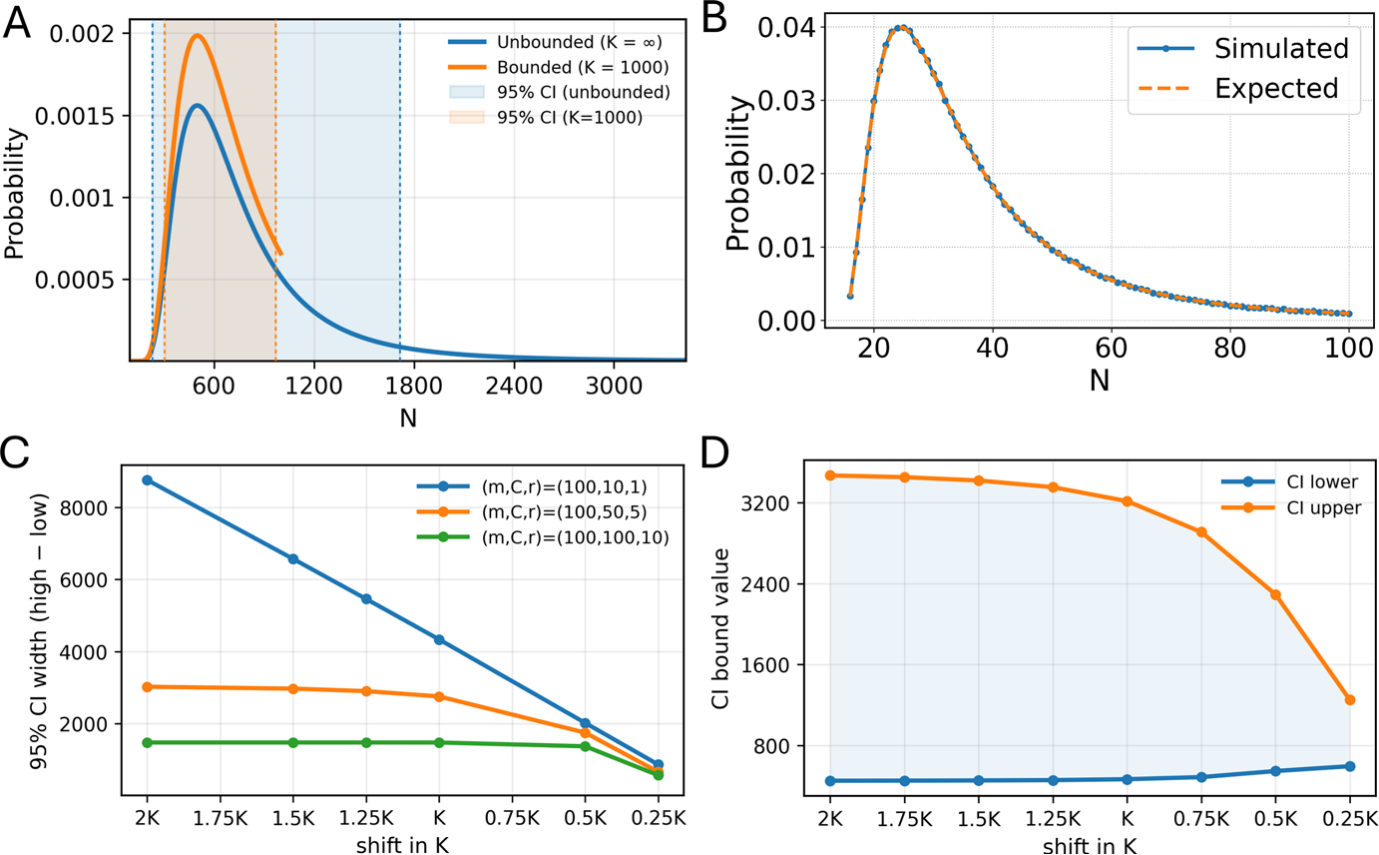
Validating and stress-testing the EHP under truncation. **(A)** For $(m,C,r)=(100,100,20)$ we compare the unbounded posterior ($K=\infty$) to a truncated posterior ($K=1000$); shading and dashed lines indicate the 95% HPD interval. Truncation removes right-tail mass beyond *K* and redistributes it over $N\le K$, yielding a more concentrated posterior and a markedly smaller upper HPD endpoint. The lower endpoint changes little because most left-tail mass already lies well below *K*. **(B)** Simulation (Monte Carlo) check of the truncated posterior for $(m,C,r,K)=(10,10,4,100)$. The near-perfect overlap confirms that the analytic EHP computation reproduces the simulation-based reference posterior over the shown range. **(C)** Sensitivity of interval width to the truncation bound. Absolute 95% HPD width is reported as *K* varies over multiplicative shifts of a baseline value $K_0=5000$ for three regimes: $(m,C,r)=(100,10,1)$ (sparse), (100, 50, 5) (intermediate), and (100, 100, 10) (informative). Width is highly *K*-dependent when recapture information is weakest (a heavy right tail), but becomes nearly insensitive when posterior mass concentrates far below the bound. **(D)** For the intermediate regime (100, 50, 5), the HPD lower and upper endpoints are plotted separately across the same *K* sweep. The lower bound remains comparatively stable, whereas the upper bound declines sharply as *K* is reduced, showing that truncation-driven changes arise primarily from constraining the right tail

Figure 2A shows the effect of truncation quantitatively for a representative case, comparing the unbounded posterior to its *K*-truncated counterpart. Truncation primarily removes right-tail mass, typically reducing the upper HPD bound while leaving the lower bound close to its unbounded value when most mass already lies well below *K*.

Throughout this study, we validated the analytic EHP calculations using Monte Carlo simulation (Appendix E). Briefly, for each candidate *N* on the evaluation grid, we simulate repeated recapture outcomes under the hypergeometric model and estimate $\widehat{P}(r_{\textrm{obs}}\mid N)$ by frequency; normalizing over *N* yields a simulation-based posterior that can be compared directly to the analytic EHP posterior. Figure 2B demonstrates close agreement between simulated and analytic posteriors.

The practical impact of truncation across regimes is summarized in Table 2. For sparse recaptures, truncation can reduce 95% HPD widths dramatically by regularizing heavy right tails and, crucially, enabling inference when the posterior is undefined for unbounded population size with $r=1$. As recapture information increases, the posterior concentrates and the influence of *K* becomes negligible, reflected by near-zero width reductions in Table 2 for larger *r*.

Sensitivity to *K* is governed by how much posterior mass lies above the proposed bound under the unbounded model. Let $p(N)\equiv P(N\mid r)$ denote the unbounded EHP (for $r>1$) and define the retained mass as

9 $$\begin{aligned} F(K)\equiv \sum _{n=N_{\min }}^{K} p(n), \qquad T(K)\equiv 1-F(K)=P(N>K\mid r) \quad , \end{aligned}$$

where *T*(*K*) is the right-tail mass excluded by truncation. The truncated posterior is $p_K(N)=p(N)/F(K)$ for $N\le K$ and 0 otherwise, so when $T(K)\approx 0$ truncation has little effect. Figures 2C–D visualize this dependence: decreasing *K* primarily contracts intervals by reducing the upper bound, with the strongest sensitivity in heavy-tailed (small-*r*) datasets and minimal sensitivity when the posterior already places negligible mass near *K*. In applications where *K* is uncertain, we recommend reporting a sensitivity sweep over a plausible range of *K* values in addition to the primary posterior summary. Because real populations are finite, introducing *K* is always conceptually valid; when *K* is used mainly to facilitate downstream calculations (Sections 2.3–3.1), it can be set sufficiently large that *T*(*K*) is negligible and the results are effectively indistinguishable from the unbounded EHP.

**Table 2:** Effect of truncating the posterior at an upper bound $K$. For the example datasets, we compute posterior summaries from the truncated posterior $P(N\mid r,K)$ (mode, mean, median, and 95% HPD interval) and report the percent reduction in HPD width relative to the corresponding untruncated EHP interval when that reference interval is defined.

(*m*, *C*, *r*)	*K*	Mode	Mean	Median	95% HPD credible interval	Decrease in width vs. no *K* (%)
$(100,\,10,\,1)$	3000	999	1606.0	1556	(473, 2963)	–
$(100,\,20,\,2)$	3000	1000	1557.4	1471	(482, 2874)	93.4
$(100,\,30,\,3)$	3000	999	1499.5	1392	(491, 2789)	70.0
$(100,\,40,\,4)$	3000	999	1442.7	1326	(501, 2693)	48.5
$(100,\,50,\,5)$	3000	999	1390.5	1274	(513, 2585)	32.1
$(100,\,60,\,6)$	3000	999	1344.1	1233	(527, 2467)	20.4
$(100,\,70,\,7)$	3000	999	1303.8	1200	(542, 2345)	12.5
$(100,\,80,\,8)$	3000	999	1269.0	1174	(558, 2228)	7.4
$(100,\,90,\,9)$	3000	999	1239.4	1153	(575, 2122)	4.3
$(100,\,100,\,10)$	3000	999	1214.2	1136	(591, 2027)	2.5
$(100,\,100,\,10)$	2800	999	1209.8	1135	(594, 2011)	3.8
$(100,\,100,\,10)$	2400	999	1193.8	1130	(604, 1946)	8.9
$(100,\,100,\,10)$	2200	999	1179.9	1124	(615, 1889)	13.5
$(100,\,100,\,10)$	2000	999	1159.6	1115	(631, 1809)	20.0
$(200,\,200,\,20)$	4000	1999	2190.9	2126	(1380, 3143)	1.9
$(300,\,300,\,30)$	6000	3000	3191.7	3125	(2205, 4306)	0.2
$(500,\,500,\,50)$	10000	4999	5187.5	5123	(3934, 6558)	0.0

### Prior specification and sensitivity

The baseline EHP used in the main analyses corresponds to a discrete uniform prior on the stated support for *N*. In the unbounded formulation, this is an improper prior $\pi (N)\propto 1$ on $\{N_{\min },N_{\min }+1,\ldots \}$; posterior propriety is then determined by the likelihood tail, as described above. In the *K*-truncated formulation, the same assumption becomes a proper uniform prior on the finite support $\{N_{\min },\ldots ,K\}$. More generally, for any prior mass function $\pi (N)$ on the admissible support, the posterior can be written as

10 $$\begin{aligned} P_{\pi }(N \mid r,m,C,K) = \frac{L(N;r,m,C)\pi (N)}{\sum _{x=N_{\min }}^{K} L(x;r,m,C)\pi (x)} \quad , \qquad N=N_{\min },\ldots ,K \quad , \end{aligned}$$

where

11 $$\begin{aligned} L(N;r,m,C) = \frac{\left( {\begin{array}{c}m\\ r\end{array}}\right) \left( {\begin{array}{c}N-m\\ C-r\end{array}}\right) }{\left( {\begin{array}{c}N\\ C\end{array}}\right) } \end{aligned}$$

is the hypergeometric likelihood kernel and $N_{\min }=m+C-r$. Thus, Eq. (10) reduces to the *K*-truncated EHP in Eq. (8) when $\pi (N)\propto 1$. The same formulation applies to the extended models by replacing *L*(*N*; *r*, *m*, *C*) with the corresponding marginal likelihood for the assumed recapture process.

For multiple independent MR events, a nonuniform prior should be applied once to the joint likelihood,

12 $$\begin{aligned} P_{\pi }(N \mid d_1,\ldots ,d_J,K) \propto \pi (N)\prod _{j=1}^{J}L_j(N) \quad , \end{aligned}$$

rather than multiplying event-wise posteriors that each already contain the same nonuniform prior. Under the uniform prior used in the main analyses, this distinction does not affect the result because the prior contributes only constants over the common support.

Prior sensitivity is governed mainly by the right tail of the likelihood. Since $L(N;r,m,C)\propto N^{-r}$ as $N\rightarrow \infty$, a prior with polynomial tail $\pi (N)\propto N^{-a}$ gives posterior tail

$$ P_{\pi }(N\mid r,m,C)\propto N^{-(r+a)} \quad . $$

Consequently, the unbounded posterior is proper when $r+a>1$, and its mean is finite when $r+a>2$. The uniform prior corresponds to $a=0$, so the unbounded EHP is proper for $r>1$ and has a finite mean only for $r>2$, as shown above. A log-uniform prior, $\pi (N)\propto 1/N$, downweights the upper tail, whereas priors that favor large *N* increase tail sensitivity. Thus, sparse-recapture cases are intrinsically sensitive in tail-dependent summaries, while high-recapture cases should be much more robust to weak prior changes.

**Fig. 3:**
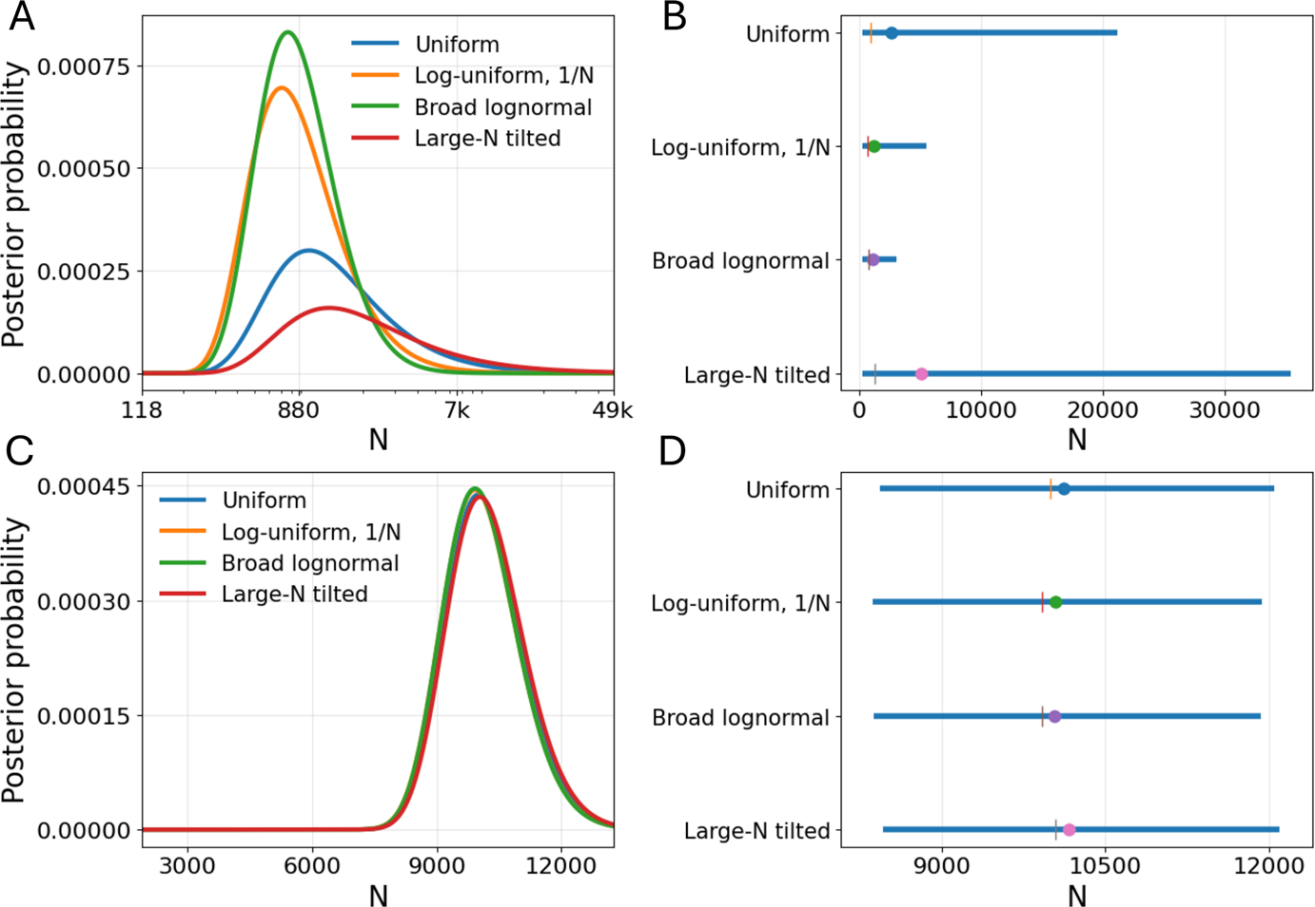
Sensitivity of EHP inference to the prior on $N$. Panels A–B show a sparse-recapture case, $(m,C,r)=(100,20,2)$ with $K=50000$, and Panels C–D show an informative case, $(m,C,r)=(1000,1000,100)$ with $K=30000$. In Panels A and C, posterior PMFs are shown under four priors: uniform, log-uniform $\pi (N)\propto 1/N$, a broad lognormal prior with log-scale center $\log (\widehat{N}_{LP})$, and a large-$N$-tilted prior proportional to $\sqrt{N/\widehat{N}_{LP}}$. Panels B and D summarize the corresponding posterior intervals; horizontal segments denote 95% HPD intervals, circles denote posterior medians, and vertical ticks denote posterior modes. Prior choice has a substantial effect on the upper tail in the sparse case but little effect when the recapture data are highly informative

We assessed this behavior by recomputing the posterior under four priors on the same finite support: the baseline uniform prior, a log-uniform prior $\pi (N)\propto 1/N$, a broad lognormal prior with log-scale center $\log (\widehat{N}_{LP})$ and log-scale standard deviation 1, and a large-*N*-tilted prior proportional to $\sqrt{N/\widehat{N}_{LP}}$ (Figure 3). For the sparse example $(m,C,r)=(100,20,2)$, prior choice substantially affects the posterior tail: the median ranges from 1091 to 5129, and the upper 95% HPD bound ranges from 3097 under the broad lognormal prior to 35419 under the large-*N*-tilted prior. This is expected because $r=2$ produces a proper but heavy-tailed likelihood under the uniform prior, so tail-dependent summaries remain sensitive to both the prior and the upper support. In contrast, for the informative example $(m,C,r)=(1000,1000,100)$, all four priors give nearly overlapping posteriors: the median ranges only from 10038 to 10163, while the lower and upper 95% HPD bounds remain within 8370–8460 and 11927–12103, respectively. These results show that EHP summaries are robust when the likelihood is informative, whereas sparse-recapture applications should report prior and *K*-sensitivity as part of the uncertainty assessment.

The relationship with profile likelihood also follows from Eq. (10). Under the same conditional hypergeometric likelihood and a uniform prior, the EHP posterior is the normalized likelihood. Therefore, the posterior mode coincides with the likelihood mode up to discreteness, and likelihood ordering is identical to posterior-mass ordering. However, EHP HPD credible intervals and profile-likelihood confidence intervals are not identical finite-sample objects: the former accumulate posterior probability mass, whereas the latter retain values of *N* whose likelihood-ratio statistic falls below a chosen cutoff. When the likelihood is strongly concentrated and the prior is locally flat, these summaries become increasingly similar; in sparse-recapture regimes, the difference remains meaningful and should be reported explicitly.

### Combining posteriors across multiple MR events

Many studies include multiple MR events (e.g., multiple pairs of marking and recapture occasions, or replicate sampling units). When these events can reasonably be treated as conditionally independent given a shared population size *N* (and when *N* is approximately constant across the events being combined), information can be aggregated by multiplying event-specific posteriors and renormalizing over a common support.

Let $d_i=(m_i,C_i,r_i)$ denote event *i*, and let $p_i(N)=P(N\mid d_i,K)$ be its *K*-truncated EHP PMF (Eq. (8)). The combined posterior is proportional to the product:

13 $$\begin{aligned} p_{\textrm{comb}}^{*}(N)=\prod _{i=1}^{J} p_i(N) \quad , \qquad N\in \{N_{\min }^{(J)},\ldots ,K\} \quad , \end{aligned}$$

where the joint lower bound is $N_{\min }^{(J)}=\max _i(m_i+C_i-r_i)$, and *K* may be taken large to minimize truncation effects.

Renormalizing yields the combined EHP:

14 $$\begin{aligned} \boxed { p_{\textrm{comb}}(N)= \frac{\displaystyle \prod _{i=1}^{J} p_i(N)}{\displaystyle \sum _{x=N_{\min }^{(J)}}^{K}\prod _{i=1}^{J} p_i(x)} \quad , \qquad N=N_{\min }^{(J)},\ldots ,K \quad . } \end{aligned}$$

On the log scale, combination is additive, $\log p_{\textrm{comb}}(N)=\textrm{const}+\sum _{i=1}^{J}\log p_i(N)$, which explains why the combined posterior typically concentrates as more events are included (Appendix D.1).

**Fig. 4:**
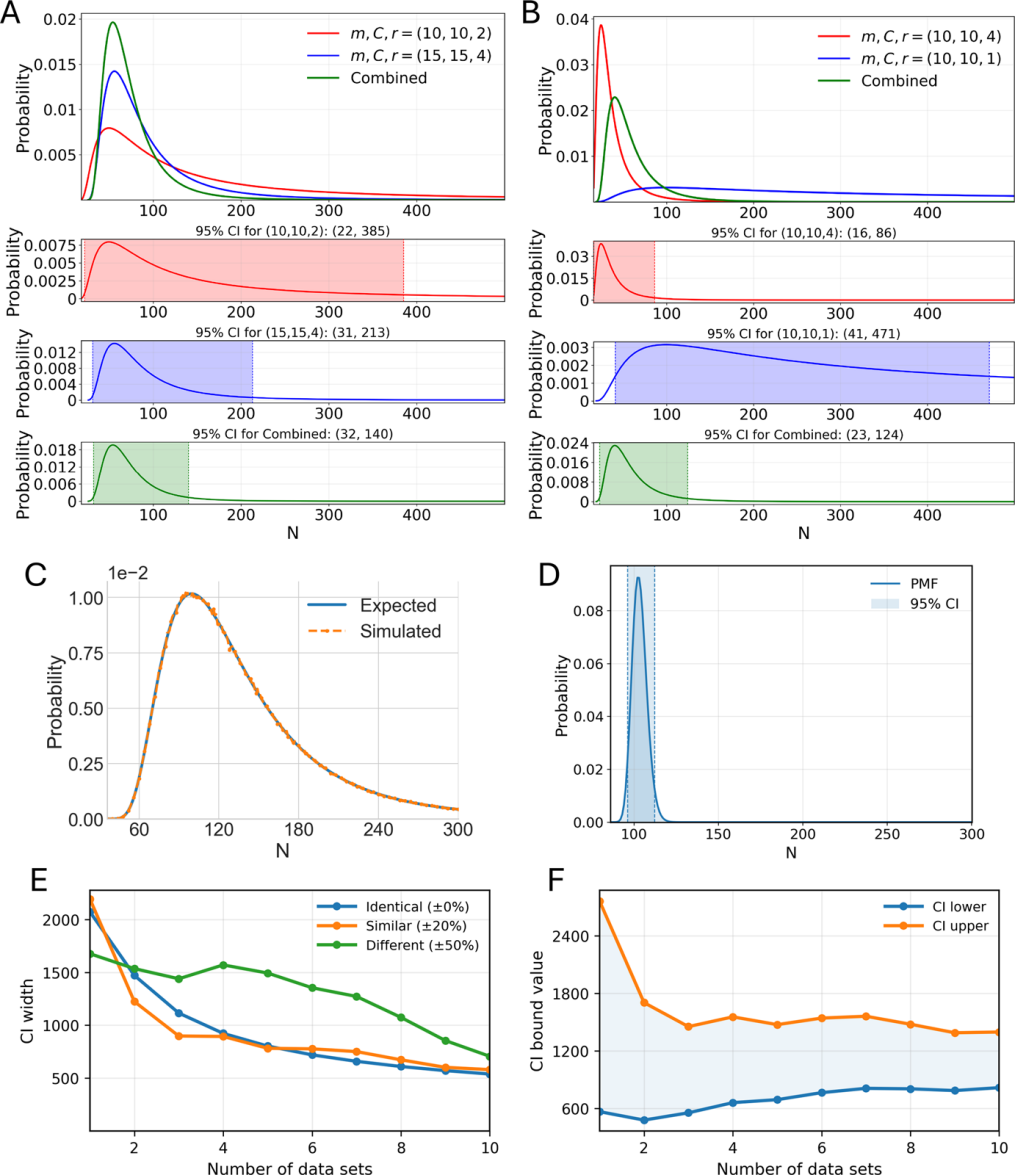
Combining posteriors across multiple mark–recapture events. **(A)** Two similar events yield strong sharpening: posteriors for $(m,C,r)=(10,10,2)$ and (15, 15, 4) overlap substantially, so their product concentrates the combined posterior and contracts the HPD interval. **(B)** Discordant information limits tightening: multiplying (10, 10, 4) (narrow) with (10, 10, 1) (heavy-tailed) yields a posterior much tighter than the weak event but potentially broader than the most informative single event, with the mode shifting to balance evidence. **(C)** Simulation validation. The analytically computed combined posterior (Expected) matches a Monte Carlo reference (Simulated) under the same hypergeometric model, supporting the product form under conditional independence. **(D)** Empirical multi-occasion example (damselfly). Combined posterior across ten capture occasions in part ($m,C,r=$ $(39,49,14),$ $(47,25,14),$ $(34,50,18),$ $(50,60,24),$ $(39,28,15),$ $(27,33,12),$ $(35,35,13),$ $(42,51,20),$ $(42,41,20),$ $(32,45,17)$), shown with upper bound $K=300$; dashed lines mark the 95% HPD interval (96, 112). Data are from (Khelifa et al. 2021).**(E)** Interval tightening as events accumulate. The 95% HPD width versus the number of events *J* for synthetic sequences of identical ($\pm 0\%$), similar ($\pm 20\%$), and different ($\pm 50\%$) events (base $(m,C,r)=100,50,5$), illustrating rapid gains under consistent conditions and slower tightening under heterogeneity. **(F)** Evolution of the 95% HPD lower and upper bounds with *J* (similar regime), showing stabilization as evidence accumulates

Figure 4A illustrates the basic effect for two events: multiplying their posterior PMFs yields a combined posterior that is more concentrated than either single-event posterior, with a visibly narrower 95% HPD interval. Figure 4B shows how this concentration depends on between-event consistency: when events support similar *N* values, the product sharpens substantially; when events are more discordant, the combined posterior remains broader and its peak shifts to balance competing evidence. These behaviors are reflected in the numerical examples in Table 3, where the reduction in interval width depends on both the information content of each event (driven by recapture counts and sample sizes) and the alignment of their posterior support.

We further validated multi-event combination by simulation. Figure 4C shows close agreement between the analytically computed combined posterior and the simulation-based estimate under the same generative model, supporting the product form in Eq. (14) under conditional independence.

A key practical benefit of Eq. (14) is the ability to integrate many modestly informative events into a highly informative combined posterior. Figure 4D provides an empirical illustration using a multi-occasion damselfly dataset: combining posteriors across events yields a tight posterior over *N* (shown with a finite upper bound *K*) and a narrow 95% HPD interval. Figures 4E–F summarize the expected tightening as the number of events increases: the 95% interval width typically decreases as additional events are included (Figure 4E), while the lower and upper bounds stabilize as evidence accumulates (Figure 4F). When events are generated under similar conditions, interval width often decreases at a rate close to the familiar $J^{-1/2}$ scaling; when events are more heterogeneous, the tightening is slower and the combined interval reflects that heterogeneity.

Unimodality of the combined posterior can be assessed exactly using the ratio of successive masses. From Eq. (8), each event-specific posterior satisfies

15 $$\begin{aligned} \frac{p_i(N+1)}{p_i(N)} = \frac{(N+1-m_i)(N+1-C_i)}{(N+1)\bigl (N+1-(m_i+C_i-r_i)\bigr )} \quad , \end{aligned}$$

for $N\ge m_i+C_i-r_i$. The corresponding ratio for the combined posterior is

16 $$\begin{aligned} \frac{p_{\textrm{comb}}(N+1)}{p_{\textrm{comb}}(N)} = \prod _{i=1}^{J}\frac{p_i(N+1)}{p_i(N)} \quad . \end{aligned}$$

The combined PMF is unimodal whenever the ratio in Eq. (16) exceeds 1 up to some index and is $\le 1$ thereafter (allowing a two-point plateau when the ratio equals 1). In practice, evaluating Eq. (16) over $N_{\min }^{(J)},\ldots ,K$ provides a simple and exact diagnostic. Empirically, and consistent with the behavior in Figures 4A–F and Table 3, the combined EHP in all examples discussed here is unimodal; more generally, we typically observe the combined posterior to remain unimodal under realistic MR event designs.

**Table 3:** Combining multiple independent mark–recapture events. For each set of independent events, we list the event-wise 95% HPD intervals and the combined posterior obtained by multiplying the event-wise posteriors and renormalizing. Combined columns report the mode, mean, median, and 95% HPD credible interval for *N* ($K=10000$).

(*m*, *C*, *r*)	95% HPD credible intervals	Combined Mode	Combined Mean	Combined Median	Combined 95% HPD credible interval
$(100,\,10,\,1)$, $(100,\,10,\,1)$	$(316,\,9081)$, $(316,\,9081)$	999	2945.8	2195	(328, 7925)
$(100,\,20,\,2)$, $(100,\,30,\,3)$	$(331,\,7918)$, $(362,\,6060)$	1000	1607.5	1337	(442, 3500)
$(100,\,50,\,5)$, $(100,\,60,\,6)$	$(449,\,3470)$, $(486,\,2918)$	999	1204.8	1127	(591, 1990)
$(50,\,50,\,25)$, $(100,\,100,\,10)$	$(85,\,128)$, $(586,\,2059)$	424	444.0	437	(335, 566)
$(10,\,10,\,1)$, $(10,\,10,\,5)$	$(25,\,8118)$, $(15,\,48)$	34	46.7	41	(22, 86)
$(100,\,10,\,1)$, $(100,\,10,\,1)$ $(100,\,10,\,1)$	$(316,\,9081)$, $(316,\,9081)$ $(316,\,9081)$	999	2261.1	1691	(356, 6089)
$(100,\,30,\,3)$, $(100,\,40,\,4)$ $(100,\,50,\,5)$	$(362,\,6060)$, $(406,\,4437)$ $(449,\,3470)$	999	1185.5	1116	(602, 1920)
$(100,\,80,\,8)$, $(100,\,90,\,9)$ $(100,\,100,\,10)$	$(544,\,2348)$, $(566,\,2183)$ $(586,\,2059)$	999	1072.1	1047	(721, 1471)
$(500,\,500,\,50)$, $(100,\,10,\,2)$ $(5,\,5,\,3)$	$(3934,\,6558)$, $(168,\,6390)$ $(7,\,37)$	4643	4799.6	4746	(3701, 5994)

## Extensions for Open Populations and Non-Ideal Recapture

In this section, we derive the EHP for the population size *N* under classical two-sample assumptions: a closed population between occasions, no loss of marked individuals, and homogeneous capture probability across individuals. In applied MR studies, two departures are especially common and can materially affect the distribution of the recapture count *r*: (i) loss of marked individuals between occasions due to mortality, permanent emigration, or tag loss, and (ii) differential capture probability between marked and unmarked individuals (for example, trap response or behavioral effects) (Touzalin et al. 2023; Cachelou et al. 2026). Here, we incorporate both effects directly at the likelihood level while preserving the discrete EHP framework, and we quantify how these mechanisms reshape the posterior and its uncertainty. We then provide tractable, data-informed models for mortality and emigration, which can be used to specify the retention component of the recapture model.

### Likelihood-based treatment of mark loss and capture heterogeneity

Under ideal assumptions, *r* follows a (central) hypergeometric distribution conditional on *N*, and the EHP is obtained via Bayes’ rule given an appropriate prior. Here, we modify the recapture likelihood to account for (i) marked–unmarked capture heterogeneity and (ii) loss of marked individuals prior to the second occasion. The resulting model preserves integer-valued data and yields a posterior that reduces to the classical EHP when the additional parameters revert to their ideal values.

#### Marked–unmarked catchability differences

To model systematic differences in capture probability between marked and unmarked individuals on the second occasion, we introduce an odds parameter $\omega$ (relative catchability). Let $w_m$ denote the capture weight for a marked individual and $w_u$ for an unmarked individual; then

17 $$\begin{aligned} \omega =\frac{w_m}{w_u} \quad , \end{aligned}$$

where $\omega >1$ indicates marked individuals are more likely to be captured (trap-happy), $\omega <1$ indicates marked individuals are less likely to be captured (trap-shy), and $\omega =1$ recovers homogeneous catchability.

#### Availability of marked individuals at recapture

Let $\phi (t)$ denote the probability that a randomly selected marked individual remains available for recapture on the second occasion, where availability encompasses survival, continued residency within the sampling frame, and retention of a detectable mark. A convenient decomposition is

18 $$\begin{aligned} \boxed { \phi (t)=\Bigl (1-\bar{d}(t)\Bigr )\Bigl (1-\overline{\textrm{em}}(t)\Bigr ) \quad , } \end{aligned}$$

where $\bar{d}(t)$ is the mean cumulative probability of death between marking and recapture, and $\overline{\textrm{em}}(t)$ is the mean cumulative probability of permanent emigration over the same interval. This product form is an independence approximation that is often adequate for short intervals; when survival and emigration are strongly coupled, $\phi (t)$ can instead be treated as a directly specified retention probability informed by auxiliary data. Sections 3.2–3.3 provide explicit expressions for $\bar{d}(t)$ and $\overline{\textrm{em}}(t)$ under parsimonious demographic and movement models.

Conditional on *m* initially marked individuals, we model the number of marked individuals that remain available at the second occasion as

19 $$\begin{aligned} M_t \mid m,\phi (t) \sim \textrm{Binomial}\!\left( m,\phi (t)\right) \quad , \end{aligned}$$

where $M_t$ is the (latent) count of available marked individuals at recapture. Decreasing $\phi (t)$ reduces the effective marked pool available for recapture and can shift and reshape the posterior for *N*.

#### Recapture likelihood conditional on availability and heterogeneity

Conditional on *N*, $m_t$ available marked individuals, and odds ratio $\omega$, the recapture count *r* follows Fisher’s noncentral hypergeometric distribution:

20 $$\begin{aligned} \boxed { P(r \mid N,m_t,C,\omega )= \frac{ \left( {\begin{array}{c}m_t\\ r\end{array}}\right) \left( {\begin{array}{c}N-m_t\\ C-r\end{array}}\right) \omega ^{r} }{ \displaystyle \sum _{j=j_{\min }}^{j_{\max }} \left( {\begin{array}{c}m_t\\ j\end{array}}\right) \left( {\begin{array}{c}N-m_t\\ C-j\end{array}}\right) \omega ^{j} } \quad , } \end{aligned}$$

with

21 $$\begin{aligned} j_{\min }=\max \!\left( 0,\,C-(N-m_t)\right) \quad ,\qquad j_{\max }=\min \!\left( C,\,m_t\right) \quad . \end{aligned}$$

When $\omega =1$, Eq. (20) reduces to the central hypergeometric likelihood used in Section 2. Together, Eqs. (19)–(20) provide a discrete likelihood that accounts for both loss of available marked individuals and marked–unmarked catchability differences.

#### Marginal likelihood under loss and heterogeneity

Because $M_t$ is typically unobserved, we marginalize over its distribution:

22 $$\begin{aligned} \boxed { P(r \mid N,m,C,\phi (t),\omega ) = \sum _{m_t=0}^{m} P(r \mid N,m_t,C,\omega )\, P(m_t \mid m,\phi (t)) \quad . } \end{aligned}$$

This marginal likelihood is fully discrete and nests the classical case: when $\phi (t)=1$ and $\omega =1$, the binomial mixture collapses to $m_t=m$, and Eq. (22) reduces to the standard hypergeometric recapture likelihood.

#### Posterior mass function for *N*

The minimum feasible population size remains

23 $$\begin{aligned} N_{\min }=m+C-r \quad , \end{aligned}$$

corresponding to the number of distinct individuals observed across both occasions. Following Section 2.2, we impose an upper bound *K* on *N* to enable computation and reflect external constraints.

Let $L(N;\,r,m,C,\phi ,\omega )=P(r \mid N,m,C,\phi ,\omega )$ denote the marginal likelihood from Eq. (22). The posterior distribution for *N* is then

24 $$\begin{aligned} \boxed { P(N \mid r,m,C,\phi (t),\omega ,K) = \frac{L(N;\,r,m,C,\phi (t),\omega )}{\displaystyle \sum _{n=N_{\min }}^{K} L(n;\,r,m,C,\phi (t),\omega )} \quad , \qquad N\in \{N_{\min },\dots ,K\} \quad . } \end{aligned}$$

**Fig. 5:**
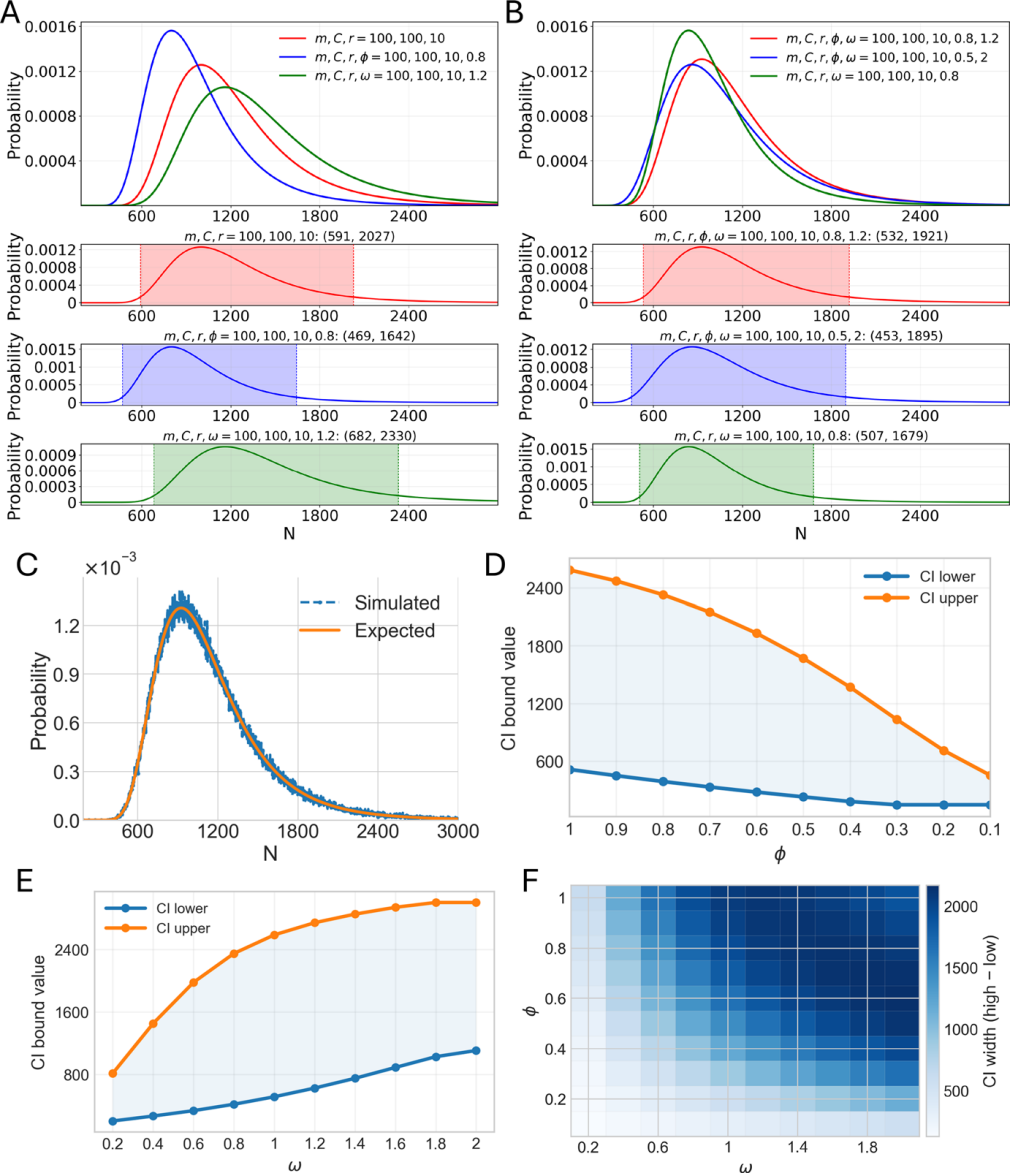
Extended EHP under mark loss and capture heterogeneity. **(A)** Single-parameter departures from $(\phi ,\omega )=(1,1)$ for $(m,C,r)=(100,100,10)$: reduced availability ($\phi =0.8$) shifts mass toward smaller $N$, while trap-happiness ($\omega =1.2$) shifts mass toward larger $N$. **(B)** Joint departures for the same dataset illustrate interactions between $\phi$ and $\omega$ and their effects on tail behavior and 95% HPD width (examples: $(\phi ,\omega )=(0.8,1.2)$, $(0.5,2)$, and $(1,0.8)$). **(C)** Simulation validation: analytic posterior (Expected) matches a Monte Carlo approximation for $(m,C,r,\phi ,\omega )=(100,100,10,0.8,1.2)$. **(D)** Sensitivity of 95% HPD endpoints to $\phi$ (holding $\omega =1$) for $(m,C,r)=(100,50,5)$. **(E)** Sensitivity of endpoints to $\omega$ (holding $\phi =1$) for the same dataset. **(F)** Two-dimensional sensitivity surface of 95% HPD interval length across the $(\phi ,\omega )$ grid for $(100,50,5)$, highlighting regimes where uncertainty inflates. In all panels $K=3000$.

An imposed upper bound *K* is needed to enable calculations. Credible intervals are obtained exactly from the posterior mass function. Figure 5A–B illustrates how $\omega$ and $\phi$ influence both posterior location and credible bounds. Figure 5C provides an implementation check by comparing an analytically computed posterior to a simulation-based approximation; the close overlap supports the correctness of both the marginalization and the noncentral hypergeometric evaluation.

When multiple two-sample datasets are available, evidence can be combined by multiplying dataset-wise marginal likelihoods. Let dataset $j\in \{1,\dots ,J\}$ contribute $(m_j,C_j,r_j)$ with (possibly) event-specific $\phi _j=\phi (t_j)$ and $\omega _j$. Define

25 $$\begin{aligned} L_{\textrm{comb}}(N)=\prod _{j=1}^{J} P(r_j \mid N,m_j,C_j,\phi _j,\omega _j) \quad , \end{aligned}$$

where each factor is given by Eq. (22), and let

26 $$\begin{aligned} N_{\min ,\textrm{comb}}=\max _{1\le j\le J}\left( m_j+C_j-r_j\right) \quad . \end{aligned}$$

The combined posterior mass function is

27 $$\begin{aligned} \boxed { {\begin{matrix} P\!\left( N \mid \{r_j,m_j,C_j\}_{j=1}^{J}, \{\phi _j,\omega _j\}_{j=1}^{J}, K\right) = \frac{ L_{\textrm{comb}}(N) }{ \displaystyle \sum _{n=N_{\min ,\textrm{comb}}}^{K} L_{\textrm{comb}}(n) } \quad , \\ \qquad N\in \{N_{\min ,\textrm{comb}},\dots ,K\} \quad . \end{matrix}} } \end{aligned}$$

With a single two-sample MR experiment, $\omega$ is generally not identifiable from the recapture count alone, because many combinations of $(N,\omega )$ can yield similar likelihood support. Consequently, $\omega$ is best treated as (i) externally informed (e.g., via behavioral studies or repeated sampling that permits estimation of trap response), or (ii) a sensitivity parameter explored over a plausible range. The same caution applies to $\phi (t)$ when mortality, emigration, and tag loss cannot be separately estimated from MR data alone. When auxiliary information is available, Sections 3.2–3.3 provide mechanistic models for constructing $\phi (t)$ from demographic and movement processes. When such information is limited, the sensitivity framework below characterizes how uncertainty in $\phi$ and $\omega$ propagates to uncertainty in *N*.

#### Sensitivity analysis

To quantify how deviations from ideal assumptions propagate into posterior uncertainty, we evaluate how the $(1-\alpha )$ credible-interval bounds depend on $\phi$ and $\omega$. Figure 5D isolates the effect of retention by plotting the lower and upper 95% credible bounds as $\phi$ varies for a representative dataset: decreasing $\phi$ shifts both bounds downward, with the upper bound often changing more strongly because the posterior mass moves toward smaller *N* while tail behavior also changes. Figure 5E analogously shows the dependence on $\omega$: increasing $\omega$ moves the credible bounds upward, reflecting the larger *N* values required to reconcile a fixed observed *r* with enhanced marked catchability. Figure 5F summarizes these effects jointly via a heat map of interval width across $(\phi ,\omega )$, highlighting regimes in which the posterior becomes substantially broader and thus less precise.

Table 4 provides complementary numerical sensitivity results for a fixed representative dataset $(m,C,r)=(200,100,20)$ (with *K* held fixed). The table shows the expected directional trends: decreasing retention ($\phi <1$ with $\omega =1$) reduces the posterior mode and median approximately proportionally (e.g., the mode decreases from $\approx 1000$ at $\phi =1$ to $\approx 500$ at $\phi =0.5$), while increasing marked catchability ($\omega >1$ with $\phi =1$) increases the inferred population size (e.g., the mode increases from $\approx 1000$ at $\omega =1$ to $\approx 1700$ at $\omega =2$). The final column in Table 4 quantifies how relative interval width can inflate markedly in some trap-happy regimes (larger $\omega$), whereas strong trap-shyness (small $\omega$) can narrow the posterior for fixed (*m*, *C*, *r*) by concentrating likelihood support on smaller *N*.

For local sensitivity, we write the marginal likelihood as

28 $$\begin{aligned} L(N;\phi ,\omega )=\sum _{m_t=0}^{m} P(r \mid N,m_t,C,\omega )\,P(m_t \mid m,\phi ) \quad , \end{aligned}$$

where $$P(m_t \mid m,\phi )=\left( {\begin{array}{c}m\\ m_t\end{array}}\right) \phi ^{m_t}(1-\phi )^{m-m_t}$$. Differentiating with respect to $\phi$ yields

29 $$\begin{aligned} \frac{\partial L}{\partial \phi } = \sum _{m_t=0}^{m} P(r \mid N,m_t,C,\omega )\,P(m_t \mid m,\phi ) \left( \frac{m_t}{\phi }-\frac{m-m_t}{1-\phi }\right) \quad . \end{aligned}$$

For the $\omega$ derivative, define the noncentral hypergeometric normalization

30 $$\begin{aligned} Z(N,m_t,C,\omega )=\sum _{j=j_{\min }}^{j_{\max }}\left( {\begin{array}{c}m_t\\ j\end{array}}\right) \left( {\begin{array}{c}N-m_t\\ C-j\end{array}}\right) \omega ^{j}, \quad \mu (N,m_t,C,\omega )=\frac{\sum _{j=j_{\min }}^{j_{\max }} j\,\left( {\begin{array}{c}m_t\\ j\end{array}}\right) \left( {\begin{array}{c}N-m_t\\ C-j\end{array}}\right) \omega ^{j}}{Z}. \end{aligned}$$

Then

31 $$\begin{aligned} \frac{\partial }{\partial \omega }P(r \mid N,m_t,C,\omega ) = P(r \mid N,m_t,C,\omega )\,\frac{r-\mu (N,m_t,C,\omega )}{\omega } \quad , \end{aligned}$$

so that

32 $$\begin{aligned} \frac{\partial L}{\partial \omega } = \sum _{m_t=0}^{m} P(m_t \mid m,\phi )\, P(r \mid N,m_t,C,\omega )\,\frac{r-\mu (N,m_t,C,\omega )}{\omega } \quad . \end{aligned}$$

Equations (29)–(32) provide score-like diagnostics for how small perturbations in $\phi$ and $\omega$ shift likelihood support across *N*. In practice, the grid-based summaries in Figure 5D–F and the numerical scenarios in Table 4 are typically more interpretable for robustness assessment, while the derivatives are useful for local approximation and for identifying regimes in which posterior precision is most sensitive to modeling assumptions.

**Table 4:** Sensitivity of EHP inference to mark loss and capture heterogeneity. Starting from $(m,C,r)=(200,100,20)$, we recompute posteriors assuming (i) only a fraction $\phi \in (0,1]$ of marked individuals remain available for recapture and (ii) biased capture modeled by Fisher’s noncentral hypergeometric distribution with odds ratio $\omega$ ($\omega >1$ favors marked; $\omega <1$ disfavors marked; $K=5000$).

$\phi$	$\omega$	Mode	Mean	Median	95% HPD credible intervals	Change in interval width vs. $\phi ,\omega =1$ (%)
1.0	1.0	999	1094.5	1061	$(704,\,1552)$	–
0.8	1.0	800	875.6	848	$(563,\,1243)$	+0.1
0.5	1.0	500	547.3	530	$(350,\,779)$	+1.1
1.0	0.8	855	931.3	904	$(619,\,1298)$	-6.4
1.0	1.5	1360	1502.5	1452	$(919,\,2187)$	+9.8
1.0	2.0	1720	1910.2	1842	$(1134,\,2822)$	+15.6
0.8	0.8	688	748.2	727	$(497,\,1043)$	-6.5
0.8	1.3	940	1034.8	1001	$(645,\,1493)$	+6.3
0.5	2.0	820	915.5	881	$(528,\,1371)$	+21.1
0.2	3.0	361	443.6	418	$(280,\,680)$	+30.5
0.7	3.0	2622	2904.1	2819	$(1660,\, 4364)$	+21.5
0.3	0.1	280	288.7	286	$(280,\,308)$	-88.2

The last column gives the percent change in relative 95% HPD width $(U-L)/\textrm{mode}$ compared with the standard case $(\phi ,\omega )=(1,1)$.

### Mortality model and mean death probability

Mortality reduces the availability of marked individuals for recapture and enters the retention probability $\phi (t)$ through the mean cumulative probability of death before potential recapture. Let *d*(*t*) denote the cumulative probability that a marked individual dies within *t* time units after marking (so $0\le d(t)\le 1$ and $d(\cdot )$ is nondecreasing). A simple approximation sometimes used when the relevant time window is short relative to lifespan is

33 $$\begin{aligned} d(t)=\frac{t}{\ell } \quad , \end{aligned}$$

where $\ell$ is the mean lifespan; more generally, *d*(*t*) can be obtained from a survival model or demographic data.

Consider a study carried out over three temporal phases: a marking period of duration $t_1$, an intervening period with no sampling of duration $t_2$, and a recapture period of duration $t_3$. Let $\tau$ denote the marking time of a randomly chosen marked individual within the marking window and let *v* denote the elapsed time within the recapture window when a potential recapture occurs. Under a uniform approximation for marking and recapture times,

$$ \tau \sim \textrm{Uniform}(0,t_1) \quad , \qquad v\sim \textrm{Uniform}(0,t_3) \quad , \qquad \text {independent.} $$

For an individual marked at time $\tau$ and potentially recaptured at elapsed time *v* during the recapture window, the time-at-risk between marking and potential recapture is

34 $$\begin{aligned} T(\tau ,v)=(t_1-\tau )+t_2+v \quad . \end{aligned}$$

Our objective is the expected death probability prior to potential recapture:

35 $$\begin{aligned} \bar{d}=\mathbb {E}\!\left[ d(T)\right] \quad . \end{aligned}$$

The joint density is

36 $$\begin{aligned} f_{\tau ,v}(\tau ,v)=\frac{1}{t_1t_3} \quad , \qquad 0\le \tau \le t_1,\ \ 0\le v\le t_3 \quad , \end{aligned}$$

so Eq. (35) becomes

37 $$\begin{aligned} \bar{d} = \frac{1}{t_1t_3}\int _{0}^{t_1}\!\!\int _{0}^{t_3} d\!\left( T(\tau ,v)\right) \,dv\,d\tau \quad . \end{aligned}$$

Substituting Eq. (34) gives the final expression:

38 $$\begin{aligned} \boxed { \bar{d} = \frac{1}{t_1t_3}\int _{0}^{t_1}\!\!\int _{0}^{t_3} d\!\left( (t_1-\tau )+t_2+v\right) \,dv\,d\tau \quad . } \end{aligned}$$

Equation (38) is general and can be evaluated numerically for any specified mortality model $d(\cdot )$. When mortality is approximately linear over the relevant window, $d(t)=\alpha t$, the integral reduces to a closed form (Appendix F):

39 $$\begin{aligned} \boxed { \bar{d}=\alpha \left( \frac{t_1}{2}+t_2+\frac{t_3}{2}\right) \quad . } \end{aligned}$$

The corresponding mean survival factor entering retention is $1-\bar{d}$, and this term can be combined with movement-driven emigration in Eq. (18) to construct $\phi (t)$ for the likelihood in Section 3.1.

### Permanent emigration and movement-driven availability

Permanent emigration (or, more generally, leaving the effective sampling frame) also reduces the availability of marked individuals for recapture. To model this effect in a way that is both interpretable and analytically tractable, we use a diffusion-based null model in which boundary encounters lead to exits at a rate determined by organism motion and the geometry of the habitat. Let *C*(*t*) denote the concentration (or availability) of organisms that are within the focal sampling region at time *t* after marking. The emigration probability is then $\textrm{em}(t)=1-C(t)$.

#### No re-entry (absorbing boundary)

If the focal region has area *A* and boundary length *b*, organisms move with mean speed *s*, and boundary crossings occur with probability *P*(*b*) upon encounter, then availability declines proportionally to the remaining fraction:

40 $$\begin{aligned} \boxed { \frac{dC}{dt}=\alpha \,C(t) \quad , \qquad \alpha =-\frac{b\,s\,f\,P(b)}{A}<0 \quad . } \end{aligned}$$

Solving yields exponential decay,

41 $$\begin{aligned} \boxed { C(t)=\exp \!\left( -\frac{b\,s\,f\,P(b)}{A}\,t\right) = e^{\alpha t} \quad , \qquad \lim _{t\rightarrow \infty }C(t)=0 \quad . } \end{aligned}$$

Here *f* is a dimensionless factor capturing geometric and behavioral effects that modulate boundary encounter rates.

#### Re-entry from a neighboring region

When organisms can leave the focal region (area $A_1$) but also re-enter from a neighboring region (area $A_2$), the net change in focal availability is the difference between outflow from region 1 and inflow from region 2. Under the same encounter-rate approximation, this gives

42 $$\begin{aligned} \boxed { \frac{dC}{dt} = -\frac{b\,s\,f\,P(b)}{A_1}\,C(t) + \frac{b\,s\,f\,P(b)}{A_2}\,[1-C(t)] \quad . } \end{aligned}$$

Writing $A_t=A_1+A_2$ and defining

$$ \beta =b\,s\,f\,P(b)\left( \frac{1}{A_1}+\frac{1}{A_2}\right) \quad , $$

the solution is

43 $$\begin{aligned} \boxed { C(t)=\frac{A_1}{A_t}+\frac{A_2}{A_t}\,e^{-\beta t} \quad , \qquad \lim _{t\rightarrow \infty }C(t)=\frac{A_1}{A_t} \quad . } \end{aligned}$$

**Fig. 6:**
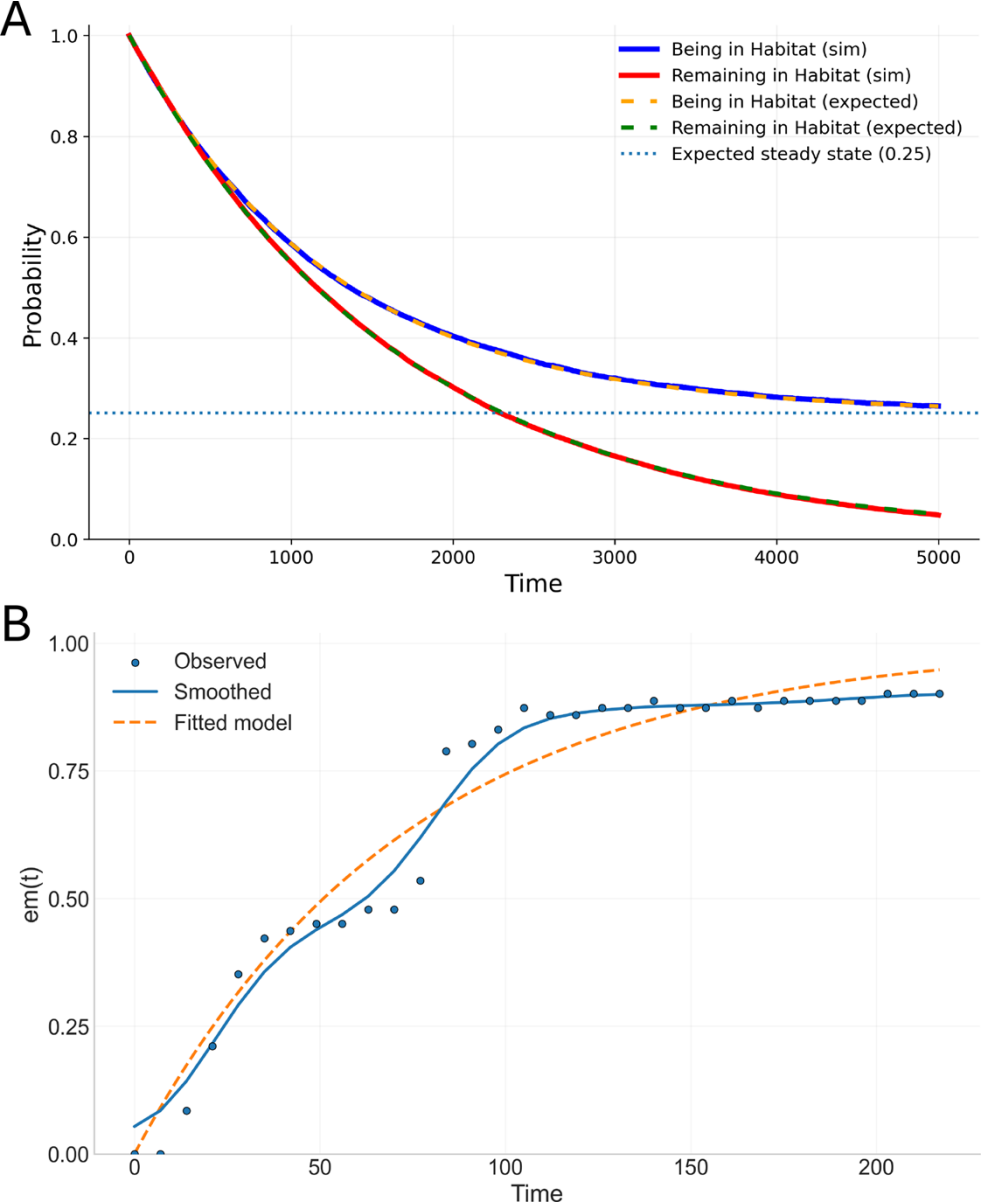
Movement-driven availability with and without re-entry, and an empirical emigration curve inferred from tracking data. **(A)** Two-region (concentric-disk) habitat illustrating how movement alone can induce time-varying availability. Individuals begin inside a focal sampling region of radius $R_1=50$ (area $A_1$) embedded within a larger accessible region of radius $R_2=100$ (total area $A_t$; surrounding area $A_2=A_t-A_1$). Boundary encounters generate transitions from focal to surrounding region at rate $k_{\textrm{out}}$ and returns at rate $k_{\textrm{in}}$ (scaling with boundary length and speed, and inversely with region area). Solid curves show stochastic simulations of the resulting two-state continuous-time Markov occupancy process (starting with $C(0)=1$), while dashed curves show analytic expectations. The *being-in-habitat* probability *C*(*t*) (blue) relaxes exponentially to the stationary occupancy $\lim _{t\rightarrow \infty }C(t)=A_1/A_t=(R_1/R_2)^2=0.25$ (dotted line), whereas the *remaining-in-habitat* probability (red) corresponds to the no-re-entry (absorbing) regime and decays as $\exp (-k_{\textrm{out}}t)$ toward zero. **(B)** Data-driven estimation of the time-dependent emigration function $em(t)=1-C(t)$ from animal tracking. Using GPS/GLS locations for Rhinoceros Auklets, a fixed sampling frame is defined as the convex hull of baseline locations; the initial cohort comprises individuals inside this hull at baseline. For each subsequent time bin, retention is the fraction of the initial cohort still observed inside the hull, and points show $\widehat{em}(t)=1-\widehat{\textrm{retention}}(t)$. The smoothed curve applies a Gaussian temporal smoother to the pointwise estimates, and the dashed line shows a simple parametric fit $em(t)\approx 1-\exp (-kt)$ as a coarse summary of dispersal/availability loss. Data from (Hipfner et al. 2024).

Figure 6A illustrates these two regimes and shows that the simulated curves agree with the analytic expressions. The curve labelled “Remaining in Habitat” corresponds to the absorbing case in Eq. (41) and decays toward zero, while the curve labelled “Being in Habitat” corresponds to Eq. (43) and converges toward the steady-state occupancy $\lim _{t\rightarrow \infty } C(t)=A_1/A_t$. In the schematic two-region geometry shown in Figure 6A, this steady-state fraction equals the area ratio (for circular regions, $(R_1/R_2)^2$), providing an interpretable geometric limit that can be checked against data.

#### Emigration probability and connection to retention

We define emigration as the complementary probability of being in the focal region:

44 $$\begin{aligned} \boxed { \textrm{em}(t)=1-C(t) \quad . } \end{aligned}$$

To obtain the mean cumulative emigration probability $\overline{\textrm{em}}(t)$ entering Eq. (18), we average $\textrm{em}(\cdot )$ over the same marking and recapture schedule used for mortality. Concretely, $\overline{\textrm{em}}$ is obtained from Eq. (38) by replacing $d(\cdot )$ with $\textrm{em}(\cdot )$, using $T(\tau ,v)$ from Eq. (34).

Figure 6B illustrates a data-driven pathway for specifying *C*(*t*) (and thus $\textrm{em}(t)$) using movement tracks. In this example, pointwise estimates derived from locations are smoothed and then fit to a parametric curve.

#### Three-dimensional extension and heterogeneous spatial structure

For a three-dimensional domain with volume *V* and surface area *S*, the same functional forms apply with the substitutions $A\rightarrow V$ and $b\rightarrow S$ (and analogously $A_1,A_2\rightarrow V_1,V_2$). In the absorbing case,

45 $$\begin{aligned} \boxed { C(t)=\exp \!\left( -\frac{S\,s\,f\,P(S)}{V}\,t\right) = e^{\alpha _{3D} t} \quad , \qquad \alpha _{3D}=-\frac{S\,s\,f\,P(S)}{V}<0 \quad . } \end{aligned}$$

With exchange between two volumes $V_1$ (focal) and $V_2$ (neighboring), writing $V_t=V_1+V_2$ gives

46 $$\begin{aligned} \boxed { C(t)=\frac{V_1}{V_t}+\frac{V_2}{V_t}\,e^{-\beta _{3D} t} \quad , \qquad \beta _{3D}=S\,s\,f\,P(S)\left( \frac{1}{V_1}+\frac{1}{V_2}\right) \quad . } \end{aligned}$$

In spatially heterogeneous settings (for example, when the population occupies multiple subregions with different emigration dynamics), a simple mixture approximation is

47 $$\begin{aligned} \textrm{em}_{\textrm{tot}}(t)= \sum _{i=1}^{n} \textrm{em}_i(t)\,\frac{N_i}{N_{\textrm{tot}}} \quad , \end{aligned}$$

where $N_i$ is the (unknown or externally estimated) subpopulation size in region *i* and $N_{\textrm{tot}}=\sum _i N_i$. This mixture provides a pragmatic way to aggregate heterogeneous emigration behavior into a single effective $\textrm{em}(t)$ for retention, while recognizing that strongly structured movement may require richer spatially explicit models when sufficient data are available.

## Comparison to Established Methods

**Fig. 7:**
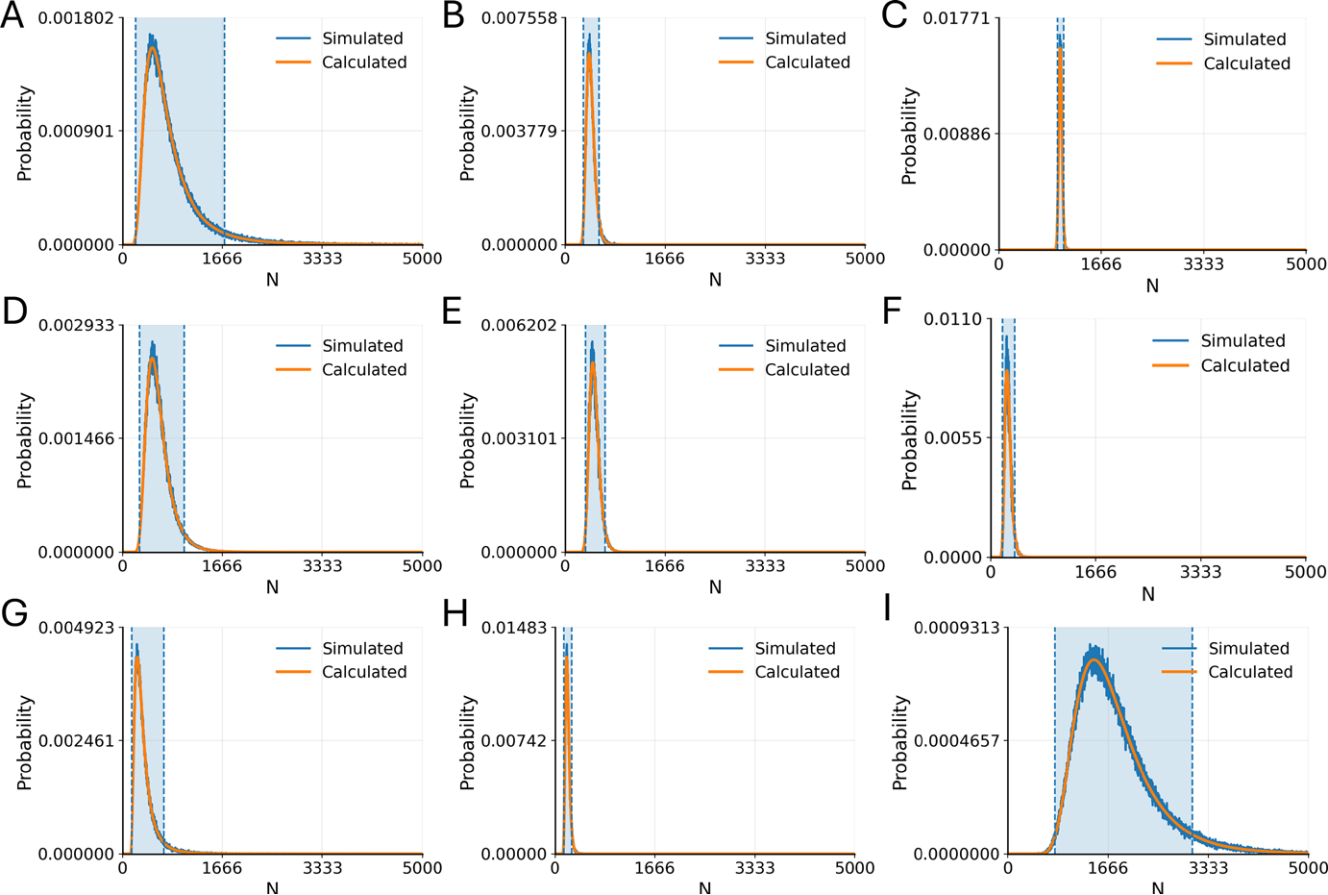
Posterior agreement between simulation-based and EHP-calculated posteriors across nine scenarios. In each panel, the simulated posterior PMF for $N$ (Monte Carlo; truncation $K=5000$) is overlaid with the EHP-calculated posterior PMF for the same scenario. Dashed vertical lines denote the 95% credible-interval endpoints and the shaded region indicates the 95% credible interval. Agreement between simulated and calculated posteriors is summarized using distributional distances (e.g., $L_1$ distance and Jensen–Shannon divergence) and interval-based metrics (width ratio WR and Jaccard overlap $J$). Panel parameter sets are: **(A–C)** $(m,C,r)=(50,50,5),(100,100,25),(500,600,300)$; **(D–F)** two- or three-event combinations with $(m,C,r)=(50,50,5)+(50,50,5)$, $(70,60,10)+(60,50,8)+(80,70,10)$, and $(50,60,8)+(30,70,10)+(20,20,4)$; **(G–I)** scenarios including capture heterogeneity with odds ratio $\omega$: $(m,C,r,\omega )=(50,60,5,0.3),(60,70,20,0.9),(100,80,10,2)$. Numerical results are reported in Table 6.

In addition to the comparisons in Table 1, we further evaluate EHP by benchmarking it against simulation-based reference posteriors and widely used capture–recapture intervals. Table 5 summarizes the competing point estimators and CI constructions, while Figure 7 and Table 6 report nine targeted stress tests spanning (i) single two-sample designs, (ii) pooled multi-event designs, and (iii) marked–unmarked catchability differences. Throughout, the Monte Carlo reference posterior is defined on a truncated integer support $\{N_{\min },\ldots ,K\}$ (here $K=5000$) and is compared to the analytic EHP posterior using distribution-level diagnostics (e.g., $L_1$ distance and Jensen–Shannon divergence) alongside interval-level diagnostics (width ratio $\textrm{WR}$ and Jaccard overlap *J*).

Panels A–C of Figure 7 illustrate why matching posterior shape matters beyond matching point estimates. In the sparse-recapture regime (Panel A; $r=5$), the posterior is strongly right-skewed and tail-dominated, so normal-approximation intervals can look precise while systematically underrepresenting uncertainty; this is reflected in Table 6 by the Chapman log CI being far too narrow (e.g., $\textrm{WR}\approx 0.41$ and $J\approx 0.41$ in Panel A). By contrast, EHP closely tracks the simulated posterior (Panel A: $\textrm{WR}\approx 1.00$, $J\approx 0.99$), expanding the 95% interval in a principled way to accommodate genuine upper-tail mass. As recapture strength increases (Panel B) and becomes strong (Panel C), both simulated and analytic posteriors concentrate near *mC*/*r*, and the gap between approximate intervals and exact/discrete approaches shrinks, consistent with the likelihood becoming sharply informative.

Panels D–F and G–I highlight two additional regimes where exact, model-aligned posteriors are of importance in practice. For multi-event designs (D–F), EHP pools information by multiplying event-wise posteriors and renormalizing, yielding uncertainty reduction that is *coherent* across events while still preserving tail behavior when each event is individually weak. Table 6 shows near-reference agreement in select cases (typically $\textrm{WR}\approx 1$ and $J\approx 0.98$–0.996). In contrast, classical multi-occasion procedures can become unstable with few occasions (e.g., Schumacher–Eschmeyer *t* intervals returning undefined or effectively unbounded limits in Panels D–F). Under heterogeneity (G–I), departures from $\omega =1$ shift both the posterior center and tail because marked and unmarked individuals are no longer sampled symmetrically; EHP captures this directly via the noncentral hypergeometric likelihood. The consequences of ignoring (or approximating) heterogeneity can be severe: for example, in Panel I ($\omega =2$), the Chao normal CI overlaps poorly with the reference posterior mass ($J\approx 0.18$), whereas EHP remains closely aligned ($J\approx 0.99$), and even the NCHG profile-likelihood CI shows nontrivial endpoint drift relative to the simulated reference.

To go beyond curated examples, Table 7 aggregates agreement metrics over 300 random scenarios (100 per category). For *single* and *heterogeneity*, we sample $m,C,r\sim \textrm{Unif}\{2,\ldots ,200\}$ subject to $r<m$, $r<C$, and $(mC/r)<1000$; for *multiple*, we pool 2–4 independent datasets with per-event $m,C,r\in$ Badia-Boher et al. (2023); Thandrayen and Baffour (2024). For each scenario, we run 2000 simulations to form a truncated reference PMF on $\{N_{\min },\ldots ,K\}$ ($K=5000$) and extract a 95% HPD-like interval via greedy expansion, matching the EHP interval definition. Under this protocol, EHP shows near-ideal calibration in *single* and *heterogeneity* settings (median $\textrm{WR}\approx 1.00$, median $J\approx 0.985$–0.988, and median endpoint error $\approx 1$–2), whereas Chapman log CIs systematically undercover (e.g., single-dataset mean endpoint error $\approx 52$) and Chao normal CIs degrade markedly under heterogeneity (mean endpoint error $\approx 143$ and $J\approx 0.50$). In the harder *multiple* category, overlaps decrease on average (median $J\approx 0.66$)—a pattern consistent with compounding Monte Carlo noise and truncation sensitivity when repeatedly multiplying weak event-wise PMFs—but EHP remains the most stable and best-aligned option among the evaluated procedures, avoiding the undefined/infinite bounds and large mismatches observed for classical multi-occasion intervals.

**Table 5:** Benchmark estimators and interval procedures used for comparison. Summary of the point estimators and 95% interval constructions evaluated against EHP, with a brief description of each method and primary references.

Method	Principle (short)	Ref.
*Point estimators*		
Lincoln–Petersen (LP) MLE	Two-sample maximum likelihood estimator under homogeneous capture; $\hat{N}$ increases with (*mC*)/*r*.	(Petersen 1896; Lincoln 1930)
Chapman estimator	Bias-corrected LP point estimator (finite-sample adjustment to reduce small-sample bias).	(Chapman 1951; Dettloff 2023)
Schnabel estimator	Multi-occasion extension of LP using pooled/weighted recapture information across sampling occasions.	(Schnabel 1938)
Schumacher–Eschmeyer estimator	Regression-based multi-occasion estimator using the linear relationship implied by capture probabilities across occasions.	(Schumacher and Eschmeyer 1943; Dettloff 2023)
Chao estimator (two-sample)	Lower-bound point estimator accounting for heterogeneity via unseen-individual correction.	(Chao 1989, 1987)
Noncentral hypergeometric (NCHG) MLE	Likelihood-based point estimate under biased sampling modeled by a noncentral hypergeometric distribution parameterized by $\omega$.	(Wallenius 1963; Liao and Rosen 2001; Fog 2008)
***CI procedures***		
Chapman log CI	Approximate CI using (approximately) normal behavior on a log scale for Chapman/LP-type estimators.	(Adams 1951)
Exact hypergeometric CI (inversion)	Exact CI obtained by inverting the hypergeometric model over *N* (bounded above by *K* when imposed).	(Dang et al. 2021; Wang 2015)
Schnabel Poisson CI	Approximate CI for the Schnabel estimator using a Poisson approximation for recapture counts.	(Friedenberg et al. 2018; Krebs 2016)
Schumacher–Eschmeyer *t* CI	Regression-based CI using a *t* distribution (requires sufficiently many capture occasions; can be unstable for small *T*).	(Schneider et al. 2000)
Chao normal CI (two-sample)	Approximate CI based on Chao variance approximation with normal-based bounds.	(Chao 1989, 1987)
NCHG profile-likelihood CI	CI obtained by profiling the NCHG likelihood over *N* (bounded above by *K* when imposed).	(Gimenez et al. 2005; Felix-Medina et al. 2015)

These baselines cover standard homogeneous-capture assumptions, multi-occasion sampling, heterogeneity corrections, and biased capture models.

**Table 6:** Quantitative comparison of interval agreement across scenarios. For each scenario (corresponding to the nine panels in Figure 7), we compare the simulation-derived reference posterior/interval with the EHP-calculated posterior and with selected benchmark estimators/intervals. Reported quantities include posterior summaries (mode, mean, and 95% interval bounds where applicable) and agreement metrics such as width ratio (WR), Jaccard overlap ($J$), and distributional distances (e.g., $L_1$ distance and Jensen–Shannon divergence) computed between simulated and calculated posteriors.

Inputs	Simulation	**EHP**	**Established methods**
Panel: A (*m*, *C*, *r*): (50, 50, 5) $\omega$: –	Mode: 510; Mean: 792.554; 95% interval: (228, 1699)	Mode: 499; Mean: 792.824; 95% interval: (232, 1704); WR: 1.00068; J: 0.99390; $L_1$: 0.043541; JS: 0.00105968	***Point estimators*** LP: 500; |10| Chapman: 432.5; |77.5| ***CI methods*** Chapman log: (224.62, 832.75) WR: 0.41341; J: 0.41018 Exact hypergeom: (243, 1466) WR: 0.83141; J: 0.83141
Panel: B (*m*, *C*, *r*): (100, 100, 25) $\omega$: –	Mode: 394; Mean: 426.194; 95% interval: (304, 564)	Mode: 399; Mean: 426.130; 95% interval: (305, 566); WR: 1.00385; J: 0.98855; $L_1$: 0.0495211; JS: 0.00102487	***Point estimators*** LP: 400; |6| Chapman: 391.346; |2.654| ***CI methods*** Chapman log: (295.53, 518.22) WR: 0.85650; J: 0.79795 Exact hypergeom: (304, 569) WR: 1.01923; J: 0.98113
Panel: C (*m*, *C*, *r*): (500, 600, 300) $\omega$: –	Mode: 1003; Mean: 1003.21; 95% interval: (953, 1053)	Mode: 999; Mean: 1003.02; 95% interval: (953, 1055); WR: 1.02; J: 0.98039; $L_1$: 0.0794445; JS: 0.00186175	***Point estimators*** LP: 1000; |3| Chapman: 999.336; |3.664| ***CI methods*** Chapman log: (950.22, 1050.99) WR: 1.00768; J: 0.95339 Exact hypergeom: (953, 1057) WR: 1.04; J: 0.96154
Panel: D (*m*, *C*, *r*): (50, 50, 5) (50, 50, 5) $\omega$: –	Mode: 498; Mean: 612.326; 95% interval: (291, 1028)	Mode: 499; Mean: 612.310; 95% interval: (293, 1029); WR: 0.99864; J: 0.99594; $L_1$: 0.0419574; JS: 0.000392133	***Point estimators*** Schnabel: 500; |2| Sch–E: 500; |2| ***CI methods*** Schnabel Poisson: (271.88, 1042.67) WR: 1.04584; J: 0.95617 Sch–E t: (NaN, NaN) WR: –; J: –
Panel: E (*m*, *C*, *r*): (70, 60, 10) (60, 50, 8) (80, 70, 10) $\omega$: –	Mode: 469; Mean: 490.573; 95% interval: (344, 661)	Mode: 460; Mean: 490.315; 95% interval: (341, 659); WR: 1.00315; J: 0.98438; L₁: 0.0585945; JS: 0.000698739	***Point estimators*** Schnabel: 457.143; |11.857| Sch–E: 465.657; |3.343| ***CI methods*** Schnabel Poisson: (316.30, 687.96) WR: 1.17242; J: 0.85294 Sch–E t: $(147.24,\infty )$ WR: –; J: –
Panel: F (*m*, *C*, *r*): (50, 60, 8) (30, 70, 10) (20, 20, 4) $\omega$: –	Mode: 263; Mean: 279.98; 95% interval: (188, 383)	Mode: 259; Mean: 280.00; 95% interval: (190, 384); WR: 0.99487; J: 0.98469; $L_1$: 0.0900811; JS: 0.00198239	***Point estimators*** Schnabel: 250; |13| Sch–E: 283.333; |20.333| ***CI methods*** Schnabel Poisson: (165.12, 398.92) WR: 1.19894; J: 0.83407 Sch–E t: $(42.67,\infty )$ WR: –; J: –
Panel: G (*m*, *C*, *r*): (50, 60, 5) $\omega$: 0.3	Mode: 252; Mean: 360.397; 95% interval: (152, 689)	Mode: 252; Mean: 360.904; 95% interval: (154, 694); WR: 1.00559; J: 0.98709; $L_1$: 0.0449175; JS: 0.00157211	***Point estimators*** Chao: 605; |353| NCHG MLE: 252; |0| ***CI methods*** Chao normal: (122.91, 1087.09) WR: 1.79548; J: 0.55695 NCHG profile: (164, 545) WR: 0.70950; J: 0.70950
Panel: H (*m*, *C*, *r*): (60, 70, 20) $\omega$: 0.9	Mode: 198; Mean: 214.36; 95% interval: (154, 284)	Mode: 199; Mean: 214.448; 95% interval: (155, 286); WR: 1.00769; J: 0.97727; $L_1$: 0.0438987; JS: 0.000869058	***Point estimators*** Chao: 211.25; |13.25| NCHG MLE: 199; |1| ***CI methods*** Chao normal: (147.15, 275.35) WR: 0.98609; J: 0.88673 NCHG profile: (156, 279) WR: 0.94615; J: 0.94615
Panel: I (*m*, *C*, *r*): (100, 80, 10) $\omega$: 2.0	Mode: 1416; Mean: 1776.76; 95% interval: (776, 3067)	Mode: 1430; Mean: 1775.92; 95	***Point estimators*** Chao: 810; |606| NCHG MLE: 1430; |14| ***CI methods*** Chao normal: (363.75, 1256.25) WR: 0.38957; J: 0.17766 NCHG profile: (824, 2843) WR: 0.88128; J: 0.88128

**Table 7:** Average interval-agreement metrics over 300 random scenarios. Across 300 randomly generated scenarios (100 per category: single two-sample datasets, multiple-dataset settings, and single-dataset heterogeneity cases), we summarize agreement between each method and the simulation reference (Monte Carlo enumeration with truncation $K=5000$) using mean/median width ratio $\textrm{WR}$, Jaccard overlap *J*, and endpoint error *E*. Endpoint error is defined as $E=\tfrac{|L-L_{\textrm{ref}}|+|U-U_{\textrm{ref}}|}{2}$ for a candidate 95% interval [*L*, *U*] relative to the reference interval $[L_{\textrm{ref}},U_{\textrm{ref}}]$.

Method	Width ratio (mean/median)	Jaccard (mean/median)	Endpoint error (mean/median)
*Single dataset*			
EHP	1.000 / 1.000	0.9780 / 0.9847	2.90 / 2.00
Chapman log CI	0.8806 / 0.9195	0.7992 / 0.8326	51.74 / 14.21
Exact hypergeometric CI (inversion)	1.048 / 1.037	0.9469 / 0.9551	8.62 / 3.00
***Multiple datasets***			
EHP	1.074 / 0.9352	0.5548 / 0.6602	117.8 / 10.5
Schnabel Poisson CI	1.873 / 1.226	0.4160 / 0.4786	128.0 / 27.61
Schumacher– Eschmeyer *t* CI	62.45 / 16.86	0.1193 / 0.05568	905.2 / 896.5
***Single dataset with heterogeneity***			
EHP	1.001 / 1.000	0.9795 / 0.9882	3.26 / 1.00
Chao normal CI	1.091 / 0.9538	0.5040 / 0.5247	143.0 / 59.34
NCHG profile-likelihood CI	0.9517 / 0.9644	0.9490 / 0.9600	17.65 / 3.00

## Discussion

We develop the EHP framework for exact inference on census population size under the classic two-sample mark–recapture design. By normalizing the hypergeometric likelihood, we obtain a posterior mass function over integer *N* that supports exact HPD credible intervals and posterior summaries without asymptotic approximations, providing a direct bridge from Lincoln–Petersen/Chapman point estimators to full-likelihood Bayesian inference (Otis et al. 1978; Seber and Schofield 2019; Dettloff 2023). This normalization also makes the baseline prior assumption explicit: the default EHP corresponds to a discrete uniform prior over the admissible support for *N*, while alternative prior choices can be incorporated by weighting the likelihood before normalization.

A central lesson is that sparse recaptures imply genuinely heavy-tailed uncertainty. When *r* is small, very large *N* values remain plausible, so the posterior right tail reflects limited information rather than a modeling artifact—precisely the regime where normal-approximation intervals can be overconfident (Otis et al. 1978; Dettloff 2023). Discreteness also reveals boundary cases: the unbounded posterior is not normalizable at $r=1$, and even when proper, some moments diverge (e.g., the mean at $r=2$), making the mode/median and HPD bounds the most stable summaries. This tail behavior also explains why prior and support sensitivity are most important in sparse-recapture regimes: upper bounds and posterior means can depend on how far the right tail is allowed to extend, whereas modes, medians, and HPD intervals are generally more stable once the likelihood is sufficiently concentrated. Because the lower endpoint is typically pinned near the feasibility limit $N_{\min }=m+C-r$ while the upper endpoint is tail-driven, increases in *r* often reduce uncertainty mainly by pulling down the upper HPD bound. Figure 1 and Table 1 underscore a design implication: datasets with the same ratio *mC*/*r* can still differ substantially in uncertainty, so improving recapture information—not merely matching a point estimate—is the dominant driver of precision. Practically, EHP intervals can serve as a planning tool if the posterior remains tail-dominated, additional effort should target higher recapture rates (e.g., stronger second-occasion effort or greater overlap) rather than simply enlarging the first sample.

The finite-*K* formulation makes regularization explicit as an interpretable ceiling on the sampling frame or ecological capacity. Truncation primarily affects the upper tail and matters only when non-negligible mass accumulates near *K* (small-*r* regimes in Figure 2); it becomes negligible once the posterior concentrates far below the bound (Table 2). Operationally, *K* can encode defensible external limits (e.g., habitat capacity or registry denominators) or be chosen large enough that excluded right-tail probability is negligible, yielding results effectively identical to the unbounded EHP. Since *K* is an explicit support/prior choice, a brief sensitivity sweep is preferable to treating it as a hidden tuning parameter. Under a diffuse, locally flat prior, the EHP mode coincides with the likelihood mode, but HPD credible intervals remain posterior-mass intervals and need not be identical to finite-sample profile-likelihood confidence intervals; the two are expected to agree most closely when the likelihood is sharply concentrated.

**Table 8:** Empirical comparison of EHP and established mark–recapture estimators. We compare EHP with established point and interval estimators using a multi-occasion damselfly mark–recapture dataset from (Khelifa et al. 2021). The five two-sample datasets used were (39, 49, 14), (47, 25, 14), (34, 50, 18), (50, 60, 24), and (39, 28, 15). For the single-event comparison, we used (47, 25, 14). For the multiple-event comparison, all five datasets were combined. EHP intervals are 95% HPD credible intervals computed with truncation $K=5000$; classical intervals are the corresponding 95% confidence intervals from the listed methods.

Method	Point estimate	95% interval
*Single event*		
EHP	mode 83; median 89; mean 92.0	(66, 125)
Chapman estimator + log-normal CI	82.2	(62.9, 107.4)
Lincoln–Petersen MLE + exact hypergeometric CI	83.9	(66, 127)
***Multiple events***		
Combined EHP	mode 110; median 111; mean 111.2	(99, 125)
Schnabel estimator + Poisson CI	104.4	(84.5, 130.8)
Schumacher–Eschmeyer estimator + *t* CI	105.6	(74.0, 184.3)

EHP also provides an exact and simple mechanism to accumulate evidence across repeated events by multiplying event-wise posteriors (equivalently, likelihoods under the common discrete-uniform support) and renormalizing, linking directly to classic combined estimators (Schnabel 1938; Seber 1965) and modern Bayesian treatments of repeated surveys (Webster and Kemp 2013). Beyond narrowing intervals, posterior multiplication pools information in the tails: multiple weak events can jointly suppress extreme-*N* support when they agree, while discordant events broaden the product posterior and shift its mode, offering a diagnostic of between-event inconsistency (Figure 4; Table 3). The empirical comparison in Table 8 illustrates the same practical behavior in the damselfly data: the single-event EHP agrees closely with exact hypergeometric inversion, while the combined EHP gives a substantially tighter interval than the Schnabel and Schumacher–Eschmeyer intervals while retaining full posterior summaries. Tightening depends on both per-event information and alignment across events: under similar conditions uncertainty can shrink rapidly with the number of events, whereas heterogeneous conditions yield slower gains and wider combined posteriors that honestly reflect variability.

Departures from ideal assumptions can be handled at the likelihood level via a retention probability $\phi (t)$ (availability of marked individuals) and a marked–unmarked catchability odds ratio $\omega$, so that closure violations and trap responses propagate directly to posterior location and tail behavior (Figure 5). Directionally, decreasing $\phi (t)$ tends to shift mass toward smaller *N*, while increasing $\omega$ tends to shift mass toward larger *N*; their interaction can inflate or contract interval width depending on how tail mass is redistributed. These parameters have clear ecological meaning and parallel mechanisms in list-based epidemiological capture–recapture, where ascertainment can differ for previously observed versus unobserved cases (Chao et al. 2001; Seber and Schofield 2019). In practice, combining modestly informative occasions can yield tight posteriors for threatened populations (Khelifa et al. 2021), while auxiliary movement data can inform time-varying availability and align *N* with the spatial definition of the target population (Hipfner et al. 2024). Calibrated uncertainty in *N* then feeds directly into downstream conservation metrics (e.g., minimum viable population targets and interpretation of $N/N_e$ ratios) (Traill et al. 2010; Jamieson and Allendorf 2012; Waples 2024), and similar stability is valuable in sparse-overlap surveillance settings such as COVID-19 burden estimation and malaria risk inference (Thandrayen and Baffour 2024; Rerolle et al. 2024).

The limitations of our method to infer population size can inform when and how it should be used. In a single two-sample dataset, $\phi (t)$ and $\omega$ are typically not identifiable without external information, so sensitivity analysis is often more defensible than point estimation. The retention decomposition relies on an independence approximation and the movement model is deliberately simplified; where survival and dispersal are themselves targets, more specialized approaches (including joint survival–dispersal models) may be required (Badia-Boher et al. 2023). Moreover, $\omega$ does not address individual-level capture heterogeneity, a known driver of bias in both closed and open designs (Pollock 1982; Pledger and Efford 1998; Gimenez et al. 2018). Finally, the product posterior assumes a shared *N* and conditional independence across events; if *N* changes over time or events are dependent, pooled inference can be overconfident unless modeled explicitly (Seber and Schofield 2019).

Overall, EHP clarifies what finite-sample data do and do not support, while offering extensions that map cleanly onto ecological and surveillance mechanisms. By making prior support, capacity constraints, repeated sampling, and departures from closure explicit in the likelihood and posterior, the framework supports reproducible inference and communicates uncertainty in a form directly usable for conservation and public-health decision making.

## Conclusion

We present the EHP framework for population-size inference from mark–recapture data. By normalizing the hypergeometric likelihood, EHP yields an exact posterior distribution for *N* and provides discrete HPD credible intervals that remain valid in finite samples under the assumed model, without relying on normal approximations. The framework incorporates ecological or sampling-frame constraints through a finite upper bound *K* and synthesizes repeated sampling events through posterior multiplication, enabling transparent accumulation of evidence across occasions. To address common field violations of ideal assumptions, we extend the likelihood to include loss of marked individuals through a retention probability $\phi (t)$ and differential catchability through an odds ratio $\omega$, and we emphasize sensitivity analysis when these quantities cannot be identified from the mark–recapture data alone. Because EHP is computationally tractable and interpretable, it can serve both as a standalone tool for data-sparse studies and as a principled baseline for more elaborate hierarchical or mechanistic extensions. We expect that these exact posterior summaries and extensions will be useful in conservation monitoring, ecological inference, and epidemiological surveillance, particularly in settings where quantifying uncertainty is as important as obtaining a point estimate.

## Data Availability

Not applicable.

## Posterior Normalization and Finiteness of the Normalizing Constant

For a two-sample mark–recapture experiment, the (hypergeometric) likelihood kernel for the recapture count *r* given population size *N* is

A.1 $$\begin{aligned} L(N;m,C,r)\;=\;\frac{\left( {\begin{array}{c}m\\ r\end{array}}\right) \left( {\begin{array}{c}N-m\\ C-r\end{array}}\right) }{\left( {\begin{array}{c}N\\ C\end{array}}\right) }, \qquad N \in \{N_{\min },N_{\min }+1,\ldots \}, \end{aligned}$$

where the minimum feasible population size is

A.2 $$\begin{aligned} N_{\min } \equiv m + C - r. \end{aligned}$$

We evaluate the normalizing series

A.3 $$\begin{aligned} Z_\infty \;\equiv \; \sum _{N=N_{\min }}^\infty L(N;m,C,r), \end{aligned}$$

which (under a discrete-uniform prior on $\{N_{\min },N_{\min }+1,\ldots \}$) is the posterior normalizing constant.

Writing the binomial coefficients in factorial form gives

A.4 $$\begin{aligned} Z_\infty = \sum _{N=N_{\min }}^\infty \frac{m!(N-m)!\,C!(N-C)!}{r!(m-r)!(C-r)!\,(N-m-C+r)!\,N!}. \end{aligned}$$

Now substitute the shift

$$ y \;=\; N - N_{\min } \;=\; N - (m+C-r), \qquad y=0,1,2,\ldots , $$

so that

$$ N-m = (C-r)+y,\quad N-C=(m-r)+y,\quad N-m-C+r=y,\quad N=(m+C-r)+y. $$

Then Eq. (A.4) becomes

A.5 $$\begin{aligned} Z_\infty = \frac{m!\,C!}{r!(m-r)!(C-r)!} \sum _{y=0}^{\infty } \frac{(y+C-r)!\,(y+m-r)!}{y!\,(y+m+C-r)!}. \end{aligned}$$

To recognize the remaining sum as a Gauss hypergeometric series, use $(y+a)! = a!\,(a+1)_y$ and $y!=(1)_y$, where $(q)_y$ is the Pochhammer symbol. With $a=C-r$ and $b=m-r$ we obtain

A.6

Gauss’s summation theorem states that, for $\Re (c-a-b)>0$,

A.7

Here $a=C-r+1$, $b=m-r+1$, $c=m+C-r+1$, so $c-a-b = r-1$. Thus the series converges (and the identity applies) if $r>1$. Applying Eq. (4) yields

A.8

Substituting Eq. (A.8) into Eq. (A.5) and simplifying with $\Gamma (n)=(n-1)!$ gives the closed form

A.9 $$\begin{aligned} Z_\infty = \sum _{N=N_{\min }}^\infty \frac{\left( {\begin{array}{c}m\\ r\end{array}}\right) \left( {\begin{array}{c}N-m\\ C-r\end{array}}\right) }{\left( {\begin{array}{c}N\\ C\end{array}}\right) } = \frac{m\,C}{r(r-1)}, \qquad 1< r \le \min (m,C). \end{aligned}$$

Therefore, the unbounded (uniform-prior) posterior is proper exactly when $r>1$.

## Behavior of the Normalizing Series when $r=1$

The finiteness of Eq. (A.4) is controlled by the large-*N* decay of the likelihood kernel

$$ L(N;m,C,r)=\frac{\left( {\begin{array}{c}m\\ r\end{array}}\right) \left( {\begin{array}{c}N-m\\ C-r\end{array}}\right) }{\left( {\begin{array}{c}N\\ C\end{array}}\right) }. $$

For fixed integers (*m*, *C*, *r*) with $0\le r\le \min (m,C)$ and $N\rightarrow \infty$, the binomial coefficients satisfy

$$ \left( {\begin{array}{c}N-m\\ C-r\end{array}}\right) =\frac{N^{\,C-r}}{(C-r)!}\left( 1+O\!\left( \frac{1}{N}\right) \right) , \qquad \left( {\begin{array}{c}N\\ C\end{array}}\right) =\frac{N^{\,C}}{C!}\left( 1+O\!\left( \frac{1}{N}\right) \right) , $$

so their ratio obeys

B.1 $$\begin{aligned} \frac{\left( {\begin{array}{c}N-m\\ C-r\end{array}}\right) }{\left( {\begin{array}{c}N\\ C\end{array}}\right) } = \frac{C!}{(C-r)!}\,N^{-r}\left( 1+O\!\left( \frac{1}{N}\right) \right) . \end{aligned}$$

Multiplying by the constant $$\left( {\begin{array}{c}m\\ r\end{array}}\right) $$ gives the asymptotic behavior

$$ L(N;m,C,r) = \left( {\begin{array}{c}m\\ r\end{array}}\right) \,\frac{C!}{(C-r)!}\,N^{-r} \left( 1+O\!\left( \frac{1}{N}\right) \right) . $$

Consequently, the normalizing series behaves like a *p*-series:

$$ Z_\infty \;=\;\sum _{N=N_{\min }}^\infty L(N;m,C,r) \;\asymp \;\sum _{N} N^{-r}. $$

Hence:

- If $r=1$, then $L(N)\sim mC\,N^{-1}$ and $Z_\infty$ diverges (harmonic divergence), so the unbounded posterior cannot be normalized under a uniform prior on $\{N_{\min },N_{\min }+1,\ldots \}$.
- If $r=0$, divergence is even stronger.
- If $r>1$, the series converges (consistent with Section 1).

The same asymptotic control also explains when posterior moments exist. Since $N\,p(N)$ is proportional to $N\,L(N)\sim N^{1-r}$, the posterior mean $\mathbb {E}[N\mid r]$ is finite only when $r>2$ (and diverges at $r=2$). In practice, when $r\le 1$ (or whenever an external upper constraint is warranted), one can impose a finite upper bound *K* and renormalize on $\{N_{\min },\ldots ,K\}$, which always yields a proper posterior.

## Posterior mean of *N*

Let *m*, *C*, *r* be nonnegative integers with $0\le r\le \min (m,C)$. The weighted mean of the distribution can be written as

C.1 $$\begin{aligned} S = \sum N \times P(N) \;=\; \frac{\sum _{N=m+C-r}^{\infty } N \times \frac{\left( {\begin{array}{c}m\\ r\end{array}}\right) \left( {\begin{array}{c}N-m\\ \,C-r\,\end{array}}\right) }{\left( {\begin{array}{c}N\\ C\end{array}}\right) }}{\frac{mC}{r(r-1)}} \end{aligned}$$

If $r\ge 3$, by using the identity

C.2 $$\begin{aligned} \left( {\begin{array}{c}N\\ C\end{array}}\right) =\frac{N}{C}\,\left( {\begin{array}{c}N-1\\ C-1\end{array}}\right) \end{aligned}$$

the summand is rewritten as

C.3 $$\begin{aligned} a_N = C\,\left( {\begin{array}{c}m\\ r\end{array}}\right) \, \frac{\left( {\begin{array}{c}N-m\\ \,C-r\,\end{array}}\right) }{\left( {\begin{array}{c}N-1\\ \,C-1\,\end{array}}\right) } \end{aligned}$$

Upon setting $k=N-m$, it follows that

C.4 $$\begin{aligned} S = C\,\left( {\begin{array}{c}m\\ r\end{array}}\right) \times \frac{r(r-1)}{m C} \times \sum _{k=C-r}^\infty \frac{\left( {\begin{array}{c}k\\ \,C-r\,\end{array}}\right) }{\left( {\begin{array}{c}k+m-1\\ \,C-1\,\end{array}}\right) } \end{aligned}$$

Next, the ratio of binomial coefficients is expressed in Pochhammer symbols and recognized as a  hypergeometric series at unity. By Gauss’s summation theorem, valid since $r\ge 3$, one obtains

C.5

and the necessary Pochhammer prefactor is likewise simplified. After routine gamma-factor manipulations, the claimed closed-form

C.6 $$\begin{aligned} S=\frac{m\,C\,(C-1)\,(m-1)}{r\,(r-1)\,(r-2)} \times \frac{r(r-1)}{m C} = \frac{(m-1)(C-1)}{r-2} \end{aligned}$$

is obtained, establishing convergence and the stated value.

## Credible Interval Construction

### Posterior PMF on the integer support

Throughout, *m* denotes the number of marked individuals in the first occasion, *C* the number captured in the second occasion, and *r* the number of marked individuals recaptured. The population size *N* is integer-valued and must satisfy

D.1 $$\begin{aligned} N \ge N_{\min } \equiv m + C - r, \end{aligned}$$

because $C-r$ individuals are observed as newly captured (unmarked) on the second occasion.

For the classical closed-population model (hypergeometric sampling), the likelihood of *r* given *N* is

D.2 $$\begin{aligned} L(N; m,C,r) = P(r \mid N) = \frac{\left( {\begin{array}{c}m\\ r\end{array}}\right) \left( {\begin{array}{c}N-m\\ C-r\end{array}}\right) }{\left( {\begin{array}{c}N\\ C\end{array}}\right) }, \qquad N = N_{\min }, N_{\min }+1, \ldots \end{aligned}$$

and the corresponding posterior mass function (PMF) is obtained by normalization over *N*. When $r>1$, Appendix A gives the exact closed-form normalizing constant

D.3 $$\begin{aligned} Z_\infty \equiv \sum _{N=N_{\min }}^\infty L(N; m,C,r) = \frac{mC}{r(r-1)}, \qquad (r>1), \end{aligned}$$

so the posterior for unbounded population size is

D.4 $$\begin{aligned} p(N) \equiv P(N \mid r,m,C) = \frac{L(N; m,C,r)}{Z_\infty }, \qquad N=N_{\min },N_{\min }+1,\ldots \quad (r>1). \end{aligned}$$

When an upper bound (environmental capacity) *K* is imposed, or when $r\le 1$ (for which the unbounded normalization diverges; see Appendix B), we use the truncated PMF

D.5 $$\begin{aligned} p_K(N) \equiv P(N \mid r,m,C,K) = \frac{L(N; m,C,r)}{\sum _{x=N_{\min }}^{K} L(x; m,C,r)}, \qquad N=N_{\min },\ldots ,K. \end{aligned}$$

More generally, if multiple independent datasets indexed by $i=1,\ldots ,J$ are available, we combine information by multiplying the normalized component PMFs on a common support:

D.6 $$\begin{aligned} p^{(J)}(N) \propto \prod _{i=1}^{J} p_i(N), \qquad N \in \{N_{\min }^{(J)}, \ldots , K\}, \end{aligned}$$

where $N_{\min }^{(J)}=\max _i (m_i+C_i-r_i)$ and *K* is a shared upper bound. The interval procedures below apply to any discrete PMF of the form *p*(*N*), $p_K(N)$, or $p^{(J)}(N)$.

### Definition of the reported $(1-\alpha )$ interval

Given a discrete PMF *q*(*N*) on integer *N* (standing for *p*, $p_K$, or $p^{(J)}$), a valid $(1-\alpha )$ interval is any set $\mathcal {I}$ such that

D.7 $$\begin{aligned} \sum _{N \in \mathcal {I}} q(N) \ge 1-\alpha . \end{aligned}$$

Because *N* is integer-valued, we report interval bounds as integers.

In the main text we focus on a *shortest contiguous* $(1-\alpha )$ interval, defined as

D.8 $$\begin{aligned} (L_\alpha ,U_\alpha ) = \arg \min _{L \le U} \left\{ U-L \;:\; \sum _{N=L}^{U} q(N) \ge 1-\alpha \right\} . \end{aligned}$$

For unimodal posteriors (as in the classical EHP posterior; see also the ratio test below), this choice yields a compact, two-sided interval with exact discrete coverage.

### Mode-expansion algorithm for unimodal posteriors

We compute $(L_\alpha ,U_\alpha )$ via a greedy *mode-expansion* procedure, which adds probability mass outward from the mode while keeping the set contiguous:

1. Compute the mode $\widehat{N}=\arg \max _N q(N)$ and initialize $L=U=\widehat{N}$ and $S=q(\widehat{N})$.
2. While $S < 1-\alpha$, compare the two adjacent probabilities $q(L-1)$ and $q(U+1)$ (when defined on the support). Add the side with the larger probability:
  $$ \text {if } q(L-1) > q(U+1)\text { then }L \leftarrow L-1;\quad \text {else }U \leftarrow U+1, $$
  and update $$S \leftarrow S + q(\text {new point})$$.
3. Output $$(L_\alpha ,U_\alpha )=(L,U)$$.

For unimodal discrete posteriors, probabilities decrease as one moves away from the mode, so the above procedure adds points in (locally) decreasing posterior order while maintaining contiguity, yielding an interval that is typically the shortest (or tied for shortest) among contiguous sets achieving mass $\ge 1-\alpha$.

### HPD-set construction (an alternative method)

When *K* is imposed (especially for $r \le 1$), or when *q*(*N*) may be less sharply unimodal, a fully general alternative is to construct a highest-posterior-density (HPD) set: sort *N* by *q*(*N*) from largest to smallest, include values until cumulative mass reaches $1-\alpha$, and then report bounds as the smallest interval spanning the selected set. Formally, let $\pi$ be a permutation such that $q(N_{\pi (1)}) \ge q(N_{\pi (2)}) \ge \cdots$ and define

D.9 $$\begin{aligned} \mathcal {S}_\alpha = \left\{ N_{\pi (1)}, \ldots , N_{\pi (M)}\right\} \quad \text {where}\quad M = \min \left\{ m:\sum _{j=1}^{m} q(N_{\pi (j)}) \ge 1-\alpha \right\} . \end{aligned}$$

We then report

D.10 $$\begin{aligned} L_\alpha = \min \mathcal {S}_\alpha ,\qquad U_\alpha = \max \mathcal {S}_\alpha . \end{aligned}$$

If the HPD set is contiguous (as is typical here), this coincides with a shortest interval. If it is not contiguous, $(L_\alpha ,U_\alpha )$ remains a conservative spanning interval that covers at least $1-\alpha$ posterior mass.

### Numerical stability and implementation notes

Direct evaluation of binomial coefficients can overflow for large *N*. We therefore compute *q*(*N*) via log-probabilities using the Gamma function identity

D.11 $$\begin{aligned} \log \left( {\begin{array}{c}n\\ k\end{array}}\right) = \log \Gamma (n+1)-\log \Gamma (k+1)-\log \Gamma (n-k+1), \end{aligned}$$

and normalize using stable log-sum-exp arithmetic. In the unbounded case ($r>1$), the exact $Z_\infty$ in Equation (D.3) avoids numerical summation to infinity. In the bounded case, normalization uses $\sum _{N=N_{\min }}^{K} L(N)$ as in Equation (D.5), and either the mode-expansion or HPD-set procedure produces an exact discrete $(1-\alpha )$ interval.

## Monte Carlo Simulation of Mark–Recapture Outcomes

This section describes the simulation procedure used to (i) generate synthetic recapture counts under the likelihood model with loss and marked–unmarked catchability differences and (ii) construct a simulation-based approximation to the posterior PMF of *N* for validation against the closed-form calculations.

### Generative model

Let *m* denote the number of individuals marked on the first occasion, *C* the number captured on the recapture occasion, and *r* the number of marked individuals recaptured. To allow for permanent loss of marked individuals between occasions (e.g., mortality and/or emigration), we model the number of marked individuals available at recapture as

E.1 $$\begin{aligned} M_t \mid m,\phi \sim \textrm{Binomial}(m,\phi (t)), \end{aligned}$$

where $\phi \in [0,1]$ is the retention (availability) probability.

Conditioned on *N* and a realized availability value $M_t=m_t$, the recapture occasion samples *C* individuals without replacement from a population of size *N* composed of $m_t$ marked and $N-m_t$ unmarked individuals. Marked and unmarked individuals may differ in catchability; we represent this via the odds (relative weight) parameter $\omega >0$. The resulting distribution of the recapture count *r* is Fisher’s noncentral hypergeometric distribution,

E.2 $$\begin{aligned} \begin{gathered} \mathbb {P}(r \mid N,m_t,C,\omega )= \frac{\left( {\begin{array}{c}m_t\\ r\end{array}}\right) \left( {\begin{array}{c}N-m_t\\ C-r\end{array}}\right) \omega ^{r}}{\sum \limits _{j=j_{\min }}^{j_{\max }}\left( {\begin{array}{c}m_t\\ j\end{array}}\right) \left( {\begin{array}{c}N-m_t\\ C-j\end{array}}\right) \omega ^{j}}, \qquad \\ j_{\min }=\max \!\bigl (0,\,C-(N-m_t)\bigr ),\,\, j_{\max }=\min (m_t,C). \end{gathered} \end{aligned}$$

The special case $\omega =1$ reduces to the classical hypergeometric likelihood (equal catchability).

Marginalizing over the unobserved $M_t$ yields the loss/heterogeneity likelihood

E.3 $$\begin{aligned} \mathbb {P}(r \mid N,m,C,\phi ,\omega ) = \sum _{m_t=0}^{m} \mathbb {P}(r \mid N,m_t,C,\omega )\left( {\begin{array}{c}m\\ m_t\end{array}}\right) \phi ^{m_t}(1-\phi )^{m-m_t}. \end{aligned}$$

In principle, Eq. (E.3) can be evaluated directly; however, we also use Monte Carlo simulation as an independent validation of both likelihood behavior and the resulting posterior PMF.

### Simulation algorithm

Fix a discrete support for *N* as $\{N_{\min },N_{\min }+1,\dots ,K\}$, where *K* is an upper truncation (when invoked) and $N_{\min }$ is chosen to be feasible. In practice, feasibility requires $N\ge \max (m,C)$; and when targeting a specific observed recapture count $r_{\textrm{obs}}$, using $N_{\min }\ge m+C-r_{\textrm{obs}}$ avoids evaluating values of *N* for which $r_{\textrm{obs}}$ is structurally impossible.

For each candidate *N* on this grid, we generate *S* independent replicates of the recapture count:

1. Draw availability: $m_t^{(s)} \sim \textrm{Binomial}(m,\phi )$ for $s=1,\dots ,S$.
2. Draw recaptures conditional on availability:
  - if $$\omega =1$$, sample $$r^{(s)} \sim \textrm{Hypergeometric}(N, m_t^{(s)}, C)$$;
  - otherwise, sample $r^{(s)} \sim \textrm{FisherNCH}\bigl (N,m_t^{(s)},C,\omega \bigr )$ with PMF given by Eq. (E.2).

The output is an empirical sample $\{r^{(s)}_N\}_{s=1}^S$ for each *N*.

### Monte Carlo likelihood and posterior normalization across *N*

Given an observed value $r_{\textrm{obs}}$, we approximate the likelihood for each *N* by the Monte Carlo frequency

E.4 $$\begin{aligned} \widehat{L}(N; r_{\textrm{obs}}) \equiv \widehat{\mathbb {P}}(r_{\textrm{obs}}\mid N,m,C,\phi ,\omega ) = \frac{1}{S}\sum _{s=1}^{S}\textbf{1}\!\left\{ r^{(s)}_N=r_{\textrm{obs}}\right\} . \end{aligned}$$

To obtain a simulation-based posterior PMF on the grid, we combine $\widehat{L}(N;r_{\textrm{obs}})$ with a prior $\pi (N)$ and normalize:

E.5 $$\begin{aligned} \widehat{p}(N \mid r_{\textrm{obs}}) = \frac{\widehat{L}(N; r_{\textrm{obs}})\,\pi (N)}{\sum \limits _{x=N_{\min }}^{K}\widehat{L}(x; r_{\textrm{obs}})\,\pi (x)}. \end{aligned}$$

When no prior information is imposed, we use a discrete uniform prior on $\{N_{\min },\dots ,K\}$, in which case $\widehat{p}(N \mid r_{\textrm{obs}})$ is proportional to $\widehat{L}(N;r_{\textrm{obs}})$.

### Implementation details and reproducibility

The simulation code is designed to be both numerically stable and computationally efficient:

- For $\omega \ne 1$, the PMF in Eq. (E.2) is computed on the log scale using precomputed $\log (i!)$ values (log-factorials) and log-binomial coefficients. The unnormalized log-weights are then normalized via a log-sum-exp transform before exponentiation, preventing overflow/underflow for moderate to large *N*.
- For a fixed *N*, the *S* availability draws $\{m_t^{(s)}\}$ take only a limited set of distinct values. The implementation groups replicates by unique $m_t$ values and, for $\omega \ne 1$, caches the PMF over *r* for each distinct $m_t$ so it is computed once per $(N,m_t)$ rather than once per replicate.
- When $\omega =1$, recaptures are sampled using a standard hypergeometric RNG, which is substantially faster than constructing the Fisher-NCH PMF.
- For Fisher-NCH sampling, after computing the support $\{r_{\min },\dots ,r_{\max }\}$ and corresponding probabilities, the implementation draws the number of occurrences of each *r* via a multinomial distribution and expands these counts to *S* samples. This reduces Python overhead compared to per-replicate sampling.
- All randomness is generated via NumPy’s default_rng with an optional fixed seed. Unless stated otherwise, reported simulation results use *S* sufficiently large to make Monte Carlo error negligible for plotted/compared PMFs.

The simulation-based posterior in Eq. (E.5) provides an external check of the analytic posterior calculations under both the classical case $(\phi =1,\omega =1)$ and the loss/heterogeneity extensions $(\phi <1\ \text {and/or}\ \omega \ne 1)$.

## Analytical Solution for Linear Mortality of Recaptured Individuals

This section provides the closed-form evaluation of the mean cumulative death probability prior to potential recapture under a linear mortality approximation. In the main paper, the expected death probability is

F.1 $$\begin{aligned} \bar{d} = \frac{1}{t_1 t_3}\int _{0}^{t_1}\int _{0}^{t_3} d\!\bigl ((t_1-\tau )+t_2+v\bigr )\, dv\, d\tau , \end{aligned}$$

where $\tau \sim \textrm{Uniform}(0,t_1)$ denotes the marking time within the marking window, $v \sim \textrm{Uniform}(0,t_3)$ denotes the elapsed time within the recapture window at which a potential recapture occurs, and the time-at-risk is $T(\tau ,v)=(t_1-\tau )+t_2+v$.

Assume mortality is approximately linear over the relevant window:

F.2 $$\begin{aligned} d(t) = \alpha t, \qquad \alpha >0. \end{aligned}$$

(As *d*(*t*) is a cumulative death probability, this linear form is best interpreted as a local/short-window approximation, i.e., valid when $\alpha t$ remains in [0, 1] over the time range of interest.)

Substituting (F.2) into (F.1) yields

F.3 $$\begin{aligned} \bar{d} = \frac{\alpha }{t_1 t_3}\int _{0}^{t_1}\int _{0}^{t_3} \bigl ((t_1-\tau )+t_2+v\bigr )\, dv\, d\tau . \end{aligned}$$

Evaluate the inner integral for fixed $\tau \in [0,t_1]$:

F.4 $$\begin{aligned} \int _{0}^{t_3}\bigl ((t_1-\tau )+t_2+v\bigr )\, dv&= \int _{0}^{t_3}\bigl ((t_1-\tau )+t_2\bigr )\, dv + \int _{0}^{t_3} v\, dv \nonumber \\&= \bigl ((t_1-\tau )+t_2\bigr )t_3 + \frac{t_3^2}{2}. \end{aligned}$$

Now integrate over $\tau \in [0,t_1]$:

F.5 $$\begin{aligned} \int _{0}^{t_1}\left[ \bigl ((t_1-\tau )+t_2\bigr )t_3 + \frac{t_3^2}{2}\right] d\tau&= t_3 \int _{0}^{t_1}\bigl ((t_1-\tau )+t_2\bigr )\, d\tau + \frac{t_1 t_3^2}{2}. \end{aligned}$$

The remaining integral is

F.6 $$\begin{aligned} \int _{0}^{t_1}\bigl ((t_1-\tau )+t_2\bigr )\, d\tau&= (t_1+t_2)t_1 - \int _{0}^{t_1}\tau \, d\tau \nonumber \\&= (t_1+t_2)t_1 - \frac{t_1^2}{2} = \frac{t_1^2}{2} + t_1 t_2. \end{aligned}$$

Substituting into (F.5) and then into (F.3), we obtain

F.7 $$\begin{aligned} \boxed { \bar{d} = \alpha \left( \frac{t_1}{2} + t_2 + \frac{t_3}{2}\right) . } \end{aligned}$$

Thus, under linear mortality, $\bar{d}$ is proportional to the mean time-at-risk, $E[T]=t_1/2+t_2+t_3/2$, reflecting that a randomly marked individual experiences on average half the remaining marking window, the full intervening gap, and half the recapture window before potential reobservation.

## Declarations

### Code Availability

The software used to run all simulations and conduct all the data analysis was written in Python 3.12 with NumPy 2.1 (Harris et al. 2020) and SciPy 1.14 (Virtanen et al. 2020). The software is available at the GitHub repository associated with this manuscript (https://github.com/Kalirad/DeepMarkRecapture ).
